# Sex Differences in Functional Connectivity Between Resting State Brain Networks in Autism Spectrum Disorder

**DOI:** 10.1007/s10803-021-05191-6

**Published:** 2021-07-16

**Authors:** Vânia Tavares, Luís Afonso Fernandes, Marília Antunes, Hugo Ferreira, Diana Prata

**Affiliations:** 1 Faculdade de Ciências, Instituto de Biofísica e Engenharia Biomédica, Universidade de Lisboa, Campo Grande, 1749-016 Lisboa, Portugal; 2 Faculdade de Medicina, Universidade de Lisboa, Lisboa, Portugal; 3 Serviço de Psiquiatria, Hospital Prof. Doutor Fernando Fonseca, EPE, IC 19, 2720-276 Amadora, Portugal; 4 Faculdade de Ciências, Centro de Estatística e Aplicações, DEIO, Universidade de Lisboa, Campo Grande 016, 1749-016 Lisboa, Portugal; 5 Department of Neuroimaging, Institute of Psychiatry, Psychology & Neuroscience, King’s College London, London, UK; 6 Centro de Investigação e Intervenção Social, Instituto Universitário de Lisboa (ISCTE-IUL), Lisboa, Portugal

**Keywords:** Functional connectivity, Resting-state networks, Autism spectrum disorder, Independent component analysis, Functional magnetic resonance imaging

## Abstract

Functional brain connectivity (FBC) has previously been examined in autism spectrum disorder (ASD) between-resting-state networks (RSNs) using a highly sensitive and reproducible hypothesis-free approach. However, results have been inconsistent and sex differences have only recently been taken into consideration using this approach. We estimated main effects of diagnosis and sex and a diagnosis by sex interaction on between-RSNs FBC in 83 ASD (40 females/43 males) and 85 typically developing controls (TC; 43 females/42 males). We found increased connectivity between the default mode (DM) and (a) the executive control networks in ASD (vs. TC); (b) the cerebellum networks in males (vs. females); and (c) female-specific altered connectivity involving visual, language and basal ganglia (BG) networks in ASD—in suggestive compatibility with ASD cognitive and neuroscientific theories.

**Supplementary Information:**

The online version contains supplementary material available at 10.1007/s10803-021-05191-6.

## Introduction

Autism spectrum disorder (ASD) is a neurodevelopmental disorder characterized by social, behavioral and cognitive impairments (American Psychiatric Association, 2013). The classical categorical system of diagnosing pervasive developmental disorders (i.e. autistic disorder, Asperger’s disorder, pervasive developmental disorder not otherwise specified, childhood disintegrative disorder, and Rett’s disorder) as found in the 4th edition of *Diagnostic and Statistical Manual of Mental Disorders* (DSM-IV-TR) (American Psychiatric Association, 2000), was collapsed into a single dimensional diagnosis of ASD in the 5th edition (DSM-5). The rationale behind this dimension collapse is that the core symptoms exhibited by individuals with ASD are shared across the previous categories, but within a severity degree spectrum.

Core symptoms exhibited by an individual with ASD consist of restricted, repetitive, and stereotyped patterns of behavior, as well as impairment in social communication and interaction. A diagnosis of ASD follows a set of criteria based on non-biological, clinically evaluated symptoms (American Psychiatric Association, 2013). There are a number of major cognitive hypotheses for ASD: the ‘Theory of Mind (ToM) dysfunction’ hypothesis (Baron-Cohen et al., 1985), the ‘executive dysfunction’ hypothesis (Ozonoff et al., 1991), the ‘weak central coherence’ hypothesis (Frith, 1989), and the ‘empathizing-systemizing’ hypothesis, also known as the ‘extreme male brain’ theory (Baron-Cohen, 2009). To master ToM, joint attention and cognitive and emotional empathy are required (Baron-Cohen et al., 1985). Impaired executive function (such as inhibition control, working memory, cognitive flexibility, and planning) is thought to aggravate non-social symptoms (Ozonoff et al., 1991). A more prominent low-level rather than high-level processing system might explain ASD individuals’ improved ability in quantitative tasks, relative to those requiring central coherence, such as visuospatial, auditory-verbal and perceptual tasks (Frith, 1989). Finally, sex differences in ASD prevalence, with the male to female ratio in ASD being 3:1 (Loomes et al., 2017), and in the behavior of typically developing controls (TC) correlate with ASD features. Namely, higher systemizing and lower empathizing ability is common in ASD (vs. TC) and in TC males (vs. TC females). As such, it is hypothesized that individuals with ASD present a shift in the ‘empathizing-systemizing’ continuum towards the systematizing ability (i.e. having a brain more similar to an ‘extreme’ TC male brain) (Baron-Cohen, 2009).

To validate and biomark cognitive-behavioral features of ASD, neuroscientific hypotheses supported by neuroimaging have also been put forward. The emerging, and putatively overarching, ‘disrupted connectivity’ (neuroscientific) hypothesis of ASD (Vasa et al., 2016) proposes that clinical symptoms exhibited by ASD individuals have their origin in the way the brain organizes and synchronizes its regions, and is well poised to account for the four above-mentioned cognitive hypotheses and other neuroscientific hypotheses of ASD, such as the ‘salience network dysfunction’ hypothesis (Toyomaki & Murohashi, 2013). This hypothesis postulates that the disrupted connectivity between the salience network (responsible for stimuli salience attribution) and the systems receiving processed stimuli information [the default mode (DM) and executive control networks] leads to social impairments in ASD. Disrupted connectivity can be tested in a resting-state functional magnetic resonance imaging (rs-fMRI) study, such as the present one. A resting-state approach is essential for an unbiased and more comprehensive perspective on brain function which is free from the influence of task-specific confounders, such as task performance differences between ASD and TC (Bressler & Menon, 2010). In this approach, functional brain connectivity (FBC) is measured as the correlation between the spontaneous activity of several brain regions and is compared between ASD and TC. More specifically, one can measure FBC within individual resting-state networks (RSNs) which resemble known spatial topographies of brain activation attributed to task approaches, for example: salience, visual, language, and DM networks (Damoiseaux et al., 2006; Shirer et al., 2012; Smith et al., 2009). Individuals with ASD, across the lifespan, have shown abnormal resting-state brain connectivity within the DM network, predominantly decreased, but also sometimes increased (Hull et al., 2017; Nair et al., 2020). The anterior salience (AS) network within-connectivity has been reported to be increased in children, but decreased in adolescents and adults (Uddin, 2015), which is consistent with the salience network dysfunction (neuroscientific) hypothesis of ASD (Toyomaki & Murohashi, 2013; Uddin & Menon, 2009). Additionally, there is some evidence for reduced long-range and increased short-range connectivity across the lifespan (Rane et al., 2015), consistent with the weak central coherence (cognitive) hypothesis of ASD. Most of these studies performed a within-network FBC analysis using a seed-based (hypothesis-based) approach.

The majority of functional connectivity studies in ASD are based on samples composed mainly, or only, of males, most likely due to the unbalanced sex ratio of ASD (Loomes et al., 2017). Nevertheless, the few studies which include both sexes have shown sex-specific differences (i.e. between ASD and TC) in regional functional connectivity, thereby providing growing evidence of an overall hyper-connectivity in females and hypo-connectivity in males, compared to TC (Alaerts et al., 2016; Lawrence et al., 2020; Smith et al., 2019; Ypma et al., 2016), albeit the opposite has also been reported once (Yang & Lee, 2018). For example, when comparing ASD with TC, the connectivity between the cerebellum and several cortical regions (i.e. the bilateral fusiform, the middle occipital, the middle frontal, the precentral gyri, the cingulate cortex, and the precuneus) was found to be increased in females and decreased in males across the lifespan (Smith et al., 2019). However, when compared to TC males, ASD males have shown *increased* connectivity between brain regions involved in mentalizing processes (i.e. the bilateral temporal-parietal junction), whereas ASD females have shown decreased connectivity (i.e. the medial prefrontal cortex, precuneus, and right temporal-parietal junction) (Yang & Lee, 2018), in a sample of adolescents. The within DM network connectivity has also been shown to be decreased across lifespan in ASD females, when compared to TC females (Ypma et al., 2016). Furthermore, when analyzing diagnosis-specific sex effects on seed-based functional connectivity in children and adolescents, ASD girls have shown increased connectivity between the posterior cingulate cortex (that belongs to the DM network) and the left posterior parietal cortex (that belongs to the central executive network) compared to ASD boys, but with no difference between sexes in TC (Lawrence et al., 2020). Interestingly, TC girls have shown decreased connectivity between the right frontoinsular cortex and the anterior cingulate cortex (that belong to the salience network) compared to TC boys, with no difference between sexes in ASD (Lawrence et al., 2020). Moreover, the within DM network connectivity has been shown to be decreased in TC males when comparing to TC females—with the decrease in connectivity associated with a poorer performance on a mentalizing task (Ypma et al., 2016) in a sample composed of children, adolescents and adults. Furthermore, the main effect of sex on within-network functional connectivity using a sample of children and adolescents with ASD and TC has been explored once, with girls showing increased connectivity within the DM network compared to boys (Olson et al., 2020).

More specifically, a few authors have examined FBC *between*-RSNs using a hypothesis-*free* approach (Bos et al., 2014; Cerliani et al., 2015; Nomi & Uddin, 2015; Oldehinkel et al., 2019; Olson et al., 2020; von dem Hagen et al., 2013), as we have done in this study. Compared to TC, ASD boys have shown decreased FBC between the executive control network and a network including the cingulate gyrus, in a sample composed of male children and young adolescents (Bos et al., 2014). FBC between salience and DM networks has also been shown to be decreased in male adults with ASD (von dem Hagen et al., 2013). When using a mixed-sex sample, ASD has shown decreased FBC between DM and precuneus (in children) and basal ganglia (BG) networks (in adolescents), whereas no differences were found in between-RSN FBC in adults (Nomi & Uddin, 2015). Increased FBC between BG and primary sensory [such as primary visual (PV), auditory and sensorimotor] networks and decreased FBC between auditory and sensorimotor networks were also shown in male children, adolescents and adults with ASD (Cerliani et al., 2015). When using a mixed-sex sample of children, adolescents and adults, the FBC between visual network and somatosensory and motor networks was shown to be decreased in ASD, whereas the FBC between cerebellar and sensory (including auditory, language, visual, and somatosensory networks) and motor networks was shown to be increased in ASD (Oldehinkel et al., 2019). Finally, in a study developed in parallel to ours, the main effect of ASD diagnosis and sex and the ASD diagnosis by sex interaction effect on the FBC between-RSNs were explored using a sample of children and adolescents (Olson et al., 2020). All effects were reported to be non-significant (i.e. after correction for multiple comparisons).

Remarkably, the above between-RSN FBC findings are inconsistent, possibly due to different subject inclusion choices, such as using: (a) mixed age groups [children, adolescents and adults Cerliani et al., 2015; Oldehinkel et al., 2019), children and adolescents (Bos et al., 2014; Olson et al., 2020), or only children or adolescents or adults (Nomi & Uddin, 2015; von dem Hagen et al., 2013)]; or (b) mixed sex groups (Nomi & Uddin, 2015; Oldehinkel et al., 2019; Olson et al., 2020) or only male subjects (Bos et al., 2014; Cerliani et al., 2015; von dem Hagen et al., 2013). Indeed, previous between-RSN FBC studies have shown differences between ASD and TC populations across lifespan to be age-dependent, in particular, showing decreased subcortico-cortical connectivity with age (Cerliani et al., 2015) and showing differences in between-RSN FBC (see above) in children and adolescents, but not in adults (Nomi & Uddin, 2015). Additionally, there is also growing evidence that FBC is influenced across the lifespan by sex, both in healthy subjects (Gong et al., 2011; Stumme et al., 2020; Zhang et al., 2016) and in individuals with ASD (Lai et al., 2017; Olson et al., 2020). Therefore, it is important that age and sex are accounted for when performing group comparisons based on FBC measures. Furthermore, only one of the previous between-RSNs studies (Olson et al., 2020), recently published, has explored if ASD-associated effects on between-RSNs FBC vary depending on the sex of the individuals.

In addition to the functional connectivity findings, task-based functional disturbances in ASD, compared to TC, have also been reported, such as: (a) decreased activation in the medial prefrontal cortex, the superior temporal sulcus, the anterior insula, the anterior cingulate cortex and the amygdala during social processing across the lifespan (Adriana Di Martino et al., 2009; Hernandez et al., 2015); (b) increased activation in BG during cognitive control in adults (Prat et al., 2016), possibly as a compensatory mechanism for cortical malfunction (Subramanian et al., 2017); and (c) a desynchronization of brain regions in language processing in adults (Dichter, 2012). Furthermore, some of the above effects have shown to be modeled by sex. In particular, the decreased activity in the posterior superior temporal sulcus during social processing is present in males, but not in females (when comparing ASD with TC in adults) (Kirkovski et al., 2016). During an empathy task using a sample of adults, ASD males have shown increased activation in the medial frontal gyrus compared to ASD females (an effect not present in the TC group); and ASD females have shown decreased activation in the midbrain and limbic regions compared to TC females (an effect not present in males) (Schneider et al., 2013).

In light of the overarching disrupted connectivity hypothesis of autism, and given the above lack of consistency in the literature, we sought to investigate: (1) the main effects of ASD (where we expect to replicate a few previous reports) and of sex on between-RSNs FBC [where we expect to replicate two studies—performed in parallel to our own, one in older TC adults (Stumme et al., 2020) and one in children and adolescents with ASD and TC (Olson et al., 2020)]; and (2) if and how sex influences ASD diagnosis effects (i.e. a diagnosis by sex interaction) on the between-RSNs FBC [attempting to corroborate work which has been performed once, in parallel and independently (Olson et al., 2020), using a sample derived partially from the same original database (ABIDE; see below)]. In the present study, we carefully considered subject eligibility and data acquisition choices to avoid confounding or noise-contributing effects of age, intelligence quotient, handedness, and eye state (open vs. closed) at scan. We compared female and male samples of children, adolescents and adults with ASD with age-matched TC and applied independent component analysis (ICA) on rs-fMRI data. ICA is a fully data-driven method able to identify brain regions that function in a temporally synchronized manner which, in rs-fMRI studies with healthy subjects, correspond to RSNs that resemble task-based functional brain activation (e.g. PV, sensorimotor and executive control networks) (Damoiseaux et al., 2006; Shirer et al., 2012; Smith et al., 2009).

## Methods

### Sample Description

Data were selected from the Autism Brain Imaging Data Exchange (ABIDE I and II, http://fcon_1000.projects.nitrc.org/indi/abide/) database (Di Martino et al., 2014) from a pool of 2226 individuals (142 ASD females, 918 ASD males, 280 TC females, and 886 TC males) using the following criteria: (a) having information regarding age at scan, handedness, eye status at scan and full scale intelligence quotient (FIQ); (b) having a FIQ higher than 70; (c) being right-handed; (d) having an anatomical T1-weighted image and an rs-fMRI with a full acquisition length with at least 150 time points and at least 300 s and near-full brain coverage; (e) both T1-weighted and rs-fMRI must be free of excessive artifacts (through a visual quality control, see Supplementary methods in the Supplementary material for more details) and rs-fMRI must be successfully registered to the T1-weighted image and to the Montreal Neurological Institute (MNI) template; and (f) having a maximum framewise displacement lower than 3 mm (corresponding to the size of one rs-fMRI voxel across the sample).

Forty-three females with ASD (the smallest group in the pool) met the above criteria. We then selected 43 ASD males, 43 TC females and 43 TC males from the pool that met the same criteria and had the best match (i.e. lowest difference possible) for age, FIQ, eye state at scan, and mean framewise displacement with the ASD females group. We further excluded 3 ASD females and 1 TC male in order to have no statistically significant difference (*p*-value < 0.05) in mean framewise displacement between age and eye state at scan between individuals with ASD and TC. Therefore, the final sample included 40 ASD females, 43 ASD males, 43 TC females and 42 TC males. Information regarding the groups, FIQ, autism diagnostic interview-revised (ADI-R), Social Responsiveness Scale, age, mean framewise displacement, and eye state at scan is shown in Table 1. For a more detailed sample description and the subject’s IDs included in the sample see the Supplementary methods and the Supplementary Tables S1 and S2 in the Supplementary material.

**Table 1:** Participants’ demographics

	ASD-F (n = 40)	ASD-M (n = 43)	TC-F (n = 43)	TC-M (n = 42)	Group comparison (*p*-value)
Age^a^ (years)	14.7 (6.6) [6.8, 38.8]	12.8 (3.2) [7.3, 20.6]	13.2 (4.2) [5.9, 27.8]	14.3 (5.0) [7.2, 31.8]	ASD vs. TD: 0.995 F vs. M: 0.635 ASD.F vs. ASD.M: 0.111 TC.F vs. TC.M: 0.294 ASD.F vs. TC.F: 0.231 ASD.M vs. TC.M: 0.123
FIQ^a^	100.8 (14.9) [74, 132]	102.8 (15.2) [72,132]	106.9 (14.1) [80, 132]	103.3 (13.9) [73, 132]	ASD vs. TD: 0.138 F vs. M: 0.677 ASD.F vs. ASD.M: 0.546 TC.F vs. TC.M: 0.233 ASD.F vs. TC.F: 0.055 ASD.M vs. TC.M: 0.868
ADI-R social^a,b^	17.4 (5.6) [7, 27]	20.1 (5.9) [9, 29]	–	–	ASD.F vs. ASD.M: 0.058
ADI-R verbal^a,b^	13.8 (4.5) [4, 23]	16.5 (4.0) [9, 24]	–	–	ASD.F vs. ASD.M: 0.010*
ADI-R RRB^a,b^	5.1 (2.4) [0, 12]	5.5 (2.6) [1, 12]	–	–	ASD.F vs. ASD.M: 0.568
Social Responsiveness Scale^a,c^	93.4 (29.9) [17,137]	93.1 (27.9) [42, 155]	19.7 (12.5) [2, 54]	22.4 (19.4) [2, 85]	ASD vs. TD: < 0.001* F vs. M: 0.701 ASD.F vs. ASD.M: 0.972 TC.F vs. TC.M: 0.231 ASD.F vs. TC.F: < 0.001* ASD.M vs. TC.M: < 0.001*
Eye state	34 O/6 C	37 O/6 C	37 O/6 C	36 O/6 C	ASD vs. TD: 0.950 F vs. M: 0.950 ASD.F vs. ASD.M: 0.892 TC.F vs. TC.M: 0.965 ASD.F vs. TC.F: 0.892 ASD.M vs. TC.M: 0.965
Mean framewise displacement (mm)	0.09 (0.05) [0.04, 0.25]	0.09 (0.05) [0.04, 0.23]	0.08 (0.04) [0.03, 0.20]	0.07 (0.05) [0.03, 0.25]	ASD vs. TD: 0.109 F vs. M: 0.669 ASD.F vs. ASD.M: 0.766 TC.F vs. TC.M: 0.718 ASD.F vs. TC.F: 0.243 ASD.M vs. TC.M: 0.268

*FIQ* mean framewise displacement, *ADI* and Social Responsiveness Scale and Chi-square test for eye state at scan, *ADI-R* autism diagnostic interview-revised, *ASD* autism spectrum disorder, *C* closed eyes, *F* female, *FIQ* full scale intelligence quotient, *M* male, *O* open eyes, *RRB* restrictive, repetitive, and stereotyped patterns of behavior

*Statistically significant at *p*-value < 0.05. Group comparisons were made with t-test for independent samples age at scan

^a^Data format: mean (standard deviation); [minimum, maximum]. Information was not available for ^b^8 ASD-F and 4 ASD-M; ^c^9 ASD-F, 12 ASD-M, 15 TC-F, and 17 TC-M participants

### Image Preprocessing

Standard preprocessing of functional data was performed using FMRIB Software Library (FSL, www.fmrib.ox.ac.uk/fsl; Smith et al., 2004) and included a temporal trimming to the first 300 s (i.e. 150 time points), removal of the first 3 volumes for signal stabilization, slice timing correction, realignment to the middle volume due to head movement effects, coregistration to the individual anatomical scan and normalization to MNI space (Tzourio-Mazoyer et al., 2002) using a non-linear full-search algorithm with 12 degrees of freedom and data spatial smoothing with a Gaussian 5 mm full-weighted at high maximum kernel. Afterwards, data was denoised using ICA-based Automatic Removal Of Motion Artifacts [ICA-AROMA (Pruim et al., 2015)], a data-driven method that identifies and removes head motion related independent components from the rs-fMRI data. Then, the denoised functional scans were high-pass filtered with a cutoff frequency of 0.01 Hz. The mean framewise displacement (i.e. head motion) was measured and compared between females and males (*p*-value = 0.234) and between individuals with ASD and TC [*p*-value = 0.036; a statistically significant effect driven by the Kennedy Krieger Institute site (KKI), *p*-value = 0.025]. Therefore, the participants from the KKI site with mean framewise displacement in the lowest/highest 5-th percentile were discarded (3 ASD females and 1 TC male). Global signal regression was not applied as it has been shown to alter short- and long-range correlations between brain regions, which might potentially introduce spurious group differences in regions where none truly exist (Murphy & Fox, 2017; Saad et al., 2012). Furthermore, it has been recently shown that ICA, as implemented herein, is an efficient method to separate global structured noise from global neural signal (Glasser et al., 2018).

### Resting-State Networks Extraction

An automatic decomposition of preprocessed functional data was computed using ICA from the FSL Multivariate Exploratory Linear Optimized Decomposition into Independent Components (MELODIC) version 3.15 tool (Beckmann et al., 2005). The whole dataset underwent a multi-session temporal concatenation analysis (with dimensionality of 20) and 20 z-scored independent maps were obtained. Then, 13 maps were identified as RSNs based on the following criteria: (a) minimal spatial overlap with vascular, ventricular and head-motion susceptible edge regions according to standard guidelines (Kelly et al., 2010); (b) a mean time course’s spectral power with a low-frequency range (0.01 ~ 0.1 Hz); and (c) a spatial distribution overlap with RSNs masks downloaded from the Functional Imaging in Neuropsychiatric Disorders (FIND) Lab at Stanford University (Shirer et al., 2012). The discarded maps are described in the Supplementary Table S3 and Supplementary Figure S2, in the Supplementary material.

### Functional Brain Connectivity and Statistical Analyses

A FBC matrix was built for each subject computing the Pearson correlation coefficient for each pair of RSNs mean time courses using FSLNets v0.6 (FMRIB Software Library). These matrices were then normalized using Fisher’s z-transformation to minimize inter-subject correlation variability. Pairwise differences between groups in the correlation coefficient were measured using a general linear model with age, mean framewise displacement, eye state at scan, and site as covariates, and inference was carried out using permutation testing (FSL randomise v2.9 (Winkler et al., 2014), 20 000 permutations) due to the limited sample size. In particular, the following effects on the between-RSN FBC were tested: (a) main effect of diagnosis (ASD vs. TC); (b) main effect of sex (females vs. males); and (c) interaction effect of diagnosis by sex. For the pairs of RSNs which a main or interaction effect was statistically significant, post hoc pairwise comparisons (e.g. ASD > TC; TC > ASD; males > females; females > males) were tested with a two-sample *t*-test. Additionally, as a complementary analysis, we also examined whether there were any sex-specific diagnosis effects (i.e. estimated the effect of diagnosis separately in males and in females) or diagnosis-specific sex effects (i.e. estimated the effect of sex in ASD and in TC, separately) on the between-RSN FBC. Results were considered statistically significant if showing a *p*-value < 0.05, corrected for multiple comparisons (i.e. multiple pairs of RSNs) with family-wise error rate (FWER).

### Correlation Analysis Between Functional Brain Connectivity and the ADI-R Score

A Pearson correlation coefficient was computed between the FBC of pairs of RSNs surviving statistical testing (described above) and the ADI-R score for social and communication functions and repetitive, restrictive, and stereotyped patterns of behavior using: (a) only the individuals with ASD (for the main effect of diagnosis); (b) males and females with ASD separately (for the main effect of sex); and (c) only females with ASD (for the female-specific effect of diagnosis). Correlations are considered statistically significant at a Bonferroni corrected (i.e. for 3 multiple comparisons) significance level of *p*-value < 0.017; or referred to as trends if only surpassing an uncorrected p-value < 0.05. No correlation analysis was conducted between the FBC of pairs of RSNs and the Social Responsiveness Scale.

## Results

### Resting-State Networks Extraction and FBC

**Fig. 1:**
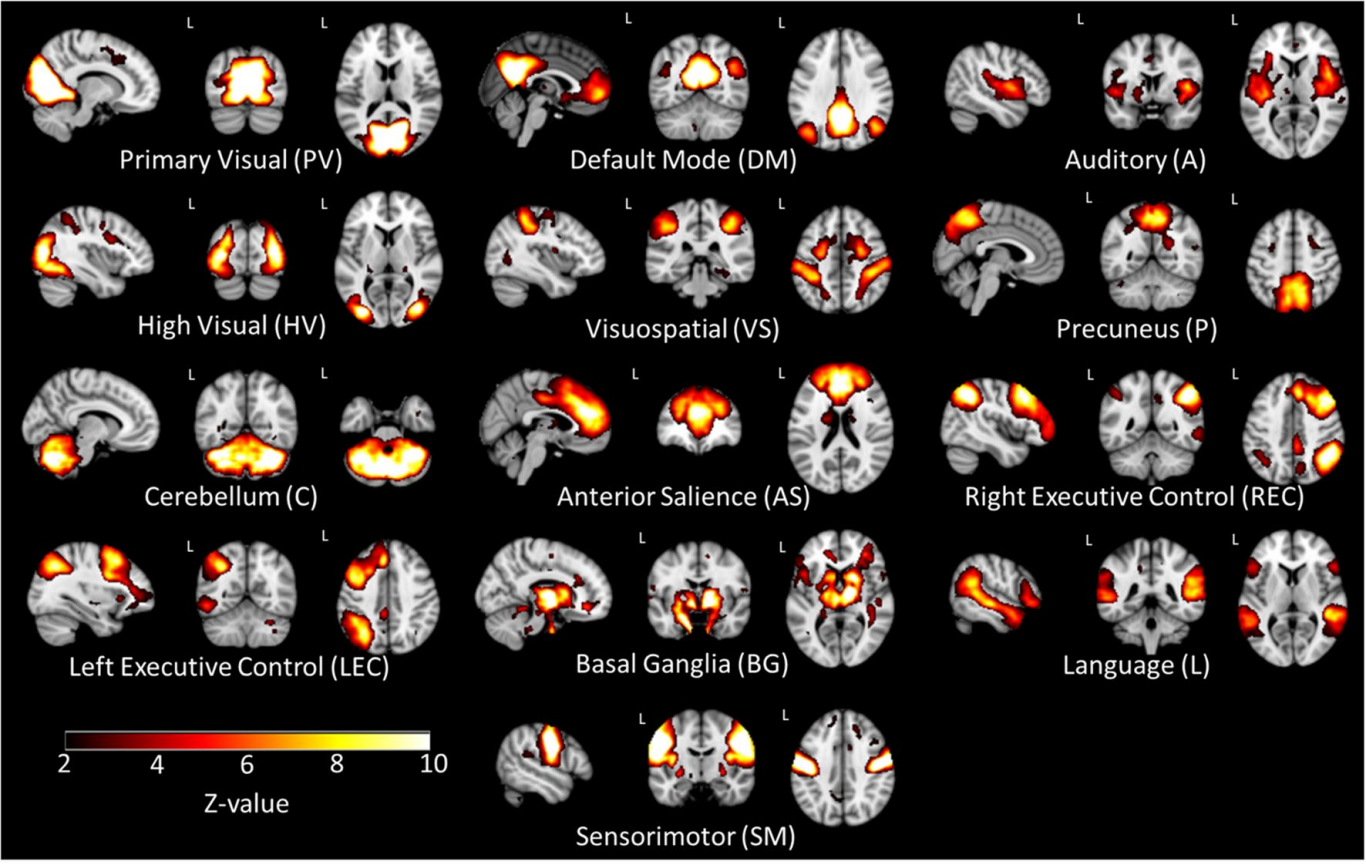
Spatial configuration of each resting-state network found by independent component analysis

From the 20 z-scored independent maps, 13 were identified as RSNs comprising AS, auditory (A), BG, cerebellum (C), DM, high visual (HV), language (L), left executive control (LEC), precuneus (P), PV, right executive control (REC), sensorimotor (SM), and visuospatial (VS) networks (Fig. 1). Seven maps were discarded as they do not resemble any of the template RSNs [(Shirer et al., 2012); Supplementary Table S3 and Supplementary Figure S2 in the Supplementary material]. Averaged FBC z-scored matrices for ASD-females, ASD-males, TC-females, and TC-males groups are depicted in Supplementary material (Supplementary Table S4 and Supplementary Figure S3).

### Between-Resting-State Networks Functional Brain Connectivity

#### Main Effect of Diagnosis

**Fig. 2:**
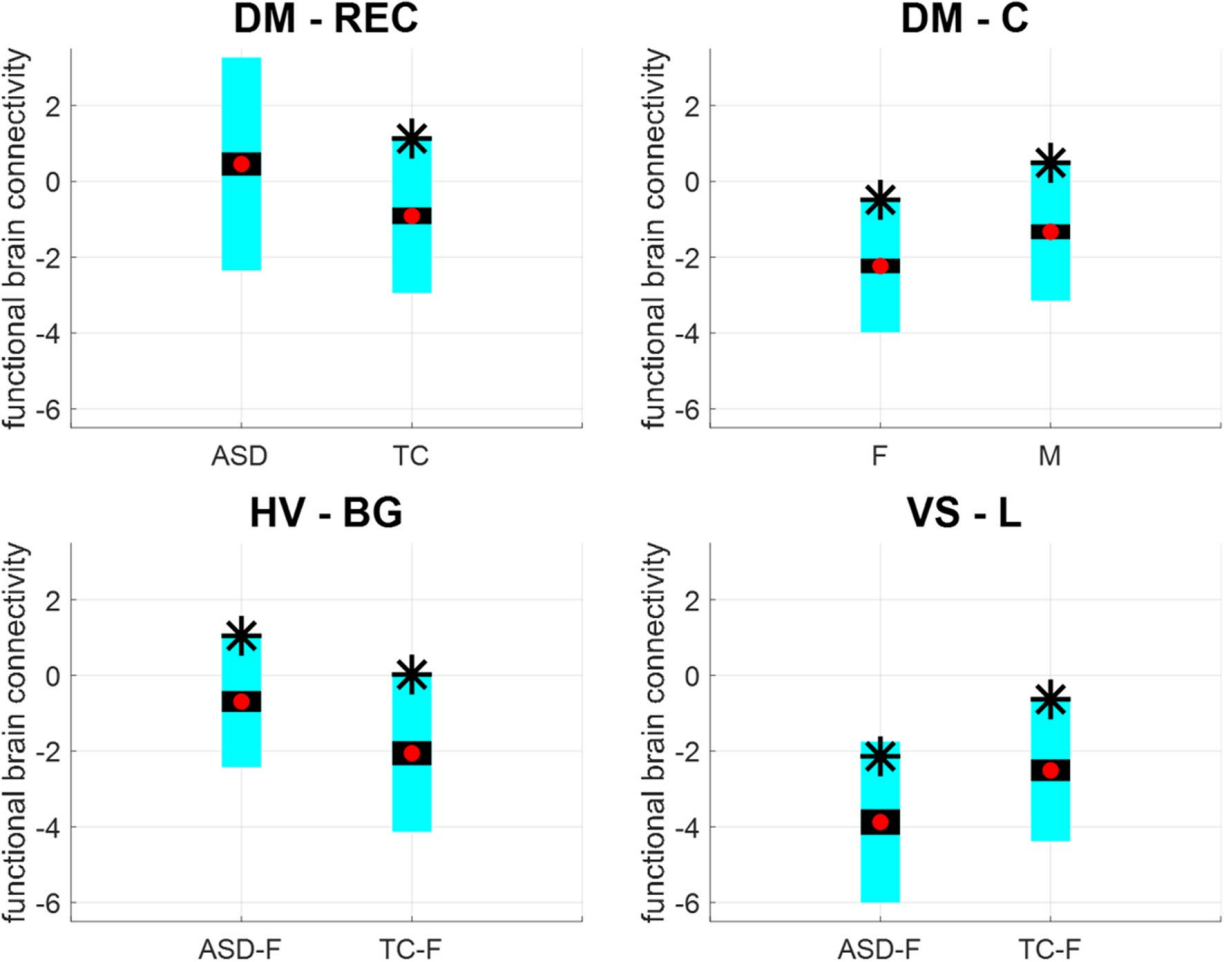
Mean z-scored Pearson correlation coefficients (red dots) per diagnostic [i.e. autism spectrum disorder (ASD) or typically developing controls (TC)] or sex [i.e. females (F) or males (M)] group for each resting-state network pair found to be different at a statistically significant level between the ASD and TC and female and male groups (uncorrected *p*-value < 0.05), and ASD-females and TC-females groups (*FWER*-corrected *p*-value < 0.05). Blue and black bars represent the standard deviation and error of the mean, respectively. In cases in which the mean was found to be significantly different from zero (tested with a Wilcoxon signed rank test with a *p*-value < 0.05), an asterisk is shown above the blue bar. *BG* basal ganglia network; *C* cerebellum network; *DM* default mode network; *HV* high visual network; *L* language network; *REC* right executive control network; *VS* visuospatial network

The main effect of diagnosis on the between-RSN FBC was statistically significant in one pair of RSN (Table 2): ‘default mode—right executive control’ (*FWER*-corrected *p*-value = 0.049), with increased correlation in ASD compared to TC (*p*-value = 0.001; Fig. 2). Furthermore, this difference was also statistically significant when correcting for all the tested RSN-pairs (*FWER*-corrected *p*-value = 0.025). See *F*- and *t*-statistic, effect size, and uncorrected and *FWER*-corrected *p*-values for every RSN pair in Supplementary material, Supplementary Table S5 and S8.

**Table 2:** Resting-state network pairs that were found to have a statistically significant effect of diagnosis (i.e. autism spectrum disorder vs. typically developing controls) or sex (females vs. males) at a statistically significant level (*FWER*-corrected *p*-value < 0.05 for main effect of diagnosis and sex (i.e. using the whole sample) and female-specific effect of diagnosis (i.e. using only females) and uncorrected *p*-value < 0.05 for post hoc comparisons)

Main effect of diagnosis	Main effect of sex	Female-specific effect of diagnosis
Default mode—right executive control (*F* = 12.12, *FWER*-corrected *p*-value = 0.049)	Default mode—cerebellum (*F* = 12.22, *FWER*-corrected *p*-value = 0.046)	*Autism spectrum disorder* > *typically developing controls* High visual—basal ganglia (*t* = 1.45, *Cohen’s d* = 0.71, *FWER*-corrected *p*-value = 0.036)
*Autism spectrum disorder* > *typical controls* Default mode—right executive control (*t* = 3.48, *Cohen’s d* = 0.56 uncorrected *p*-value = 0.001, *FWER*-corrected *p*-value = .025)	*Males* > *females* Default mode—cerebellum (*t* = 0.96, *Cohen’s d* = 0.51, uncorrected *p*-value = 0.001, *FWER*-corrected *p*-value = 0.024)	*Typically developing controls* > *autism spectrum disorder* Visuospatial—language (*t* = 3.12, *Cohen’s d* = 0.68, *FWER*-corrected *p*-value = 0.031)

#### Main Effect of Sex

The main effect of sex on the between-RSN FBC was statistically significant in one pair of RSNs (Table 2): ‘default mode—cerebellum’ (*FWER*-corrected *p*-value = 0.046), with increased correlation in males compared to females (*p*-value = 0.001; Fig. 2). Furthermore, this difference was also statistically significant when correcting for all the tested RSNs-pairs (*FWER*-corrected *p*-value = 0.024). See *F*- and *t*-statistic, effect size, and uncorrected and *FWER*-corrected *p*-values for every RSNs pair in Supplementary material, Supplementary Table S6 and S9.

#### Diagnosis by Sex Interaction

The diagnosis by sex interaction effect on the between-RSNs FBC was not statistically significant in any pair of RSNs. See *F*-statistic and uncorrected and *FWER*-corrected *p*-values for every RSNs pair in Supplementary material, Supplementary Table S7.

#### Sex-Specific Effect of Diagnosis

The female-specific effect of diagnosis on the between-RSN FBC was statistically significant in two pairs of RSNs (Table 2): (a) ‘high visual—basal ganglia’, with increased correlation in ASD compared to TC (*FWER*-corrected *p*-value = 0.036; Fig. 2); and (b) ‘visuospatial—language’, with decreased correlation in ASD compared to TC (*FWER*-corrected *p*-value = 0.031; Fig. 2). The male-specific effect of diagnosis on the between-RSNs FBC was not statistically significant in any pair of RSNs. See *t*-statistic, effect size, and uncorrected and *FWER*-corrected *p*-values for every RSNs pair in Supplementary material, Supplementary Table S10 and S11.

#### Diagnosis-Specific Effect of Sex

An ASD- or TC-specific effect of sex on the between-RSNs FBC was not statistically significant in any pair of RSNs. See *t*-statistic, effect size, and uncorrected and *FWER*-corrected *p*-values for every RSNs pair in Supplementary material, Supplementary Table S12 and S13.

### Correlation Analysis Between Functional Brain Connectivity and the ADI-R Score

The correlation analysis between the FBC of pairs of RSNs and the ADI-R scores were not statistically significant in any pair of RSNs. See Table 3 for full statistics.

**Table 3:** Correlation (i.e. *Pearson* r) between the functional brain connectivity (FBC) of pairs of resting-state networks (RSNs) and the ADI-R score for social and communication (verbal) functions and repetitive, restrictive and stereotyped patterns of behaviors (RRB)

Pairs of resting-state networks	ADI-R Social	ADI-R Verbal	ADI-R RRB
Default mode—right executive control^a^	Male + female: *r* = − 0.10, *p*-value = 0.426	Male + female *r* = − 0.09, *p*-value = 0.472	Male + female *r* = − 0.00, *p*-value = 0.972
Default mode—cerebellum^b^	Male: *r* = 0.23, *p*-value = 0.168 Female: *r* = 0.16, *p*-value = 0.401	Male: *r* = 0.11, *p*-value = 0.518 Female: *r* = 0.14, *p*-value = 0.444	Male: *r* = 0.04, *p*-value = 0.834 Female: *r* =0 .27, *p*-value = 0.150
High visual—basal ganglia^c^	Female: *r* = − 0.2, *p*-value = 0.906	Female: *r* = − 0.11, *p*-value = 0.570	Female: *r* = 0.18, *p*-value = 339
Visuospatial—language^c^	Female: *r* = − 0.08, *p*-value = 0.680	Female: *r* = − 0.8, *p*-value = 0.656	Female: *r* = − 0.05, *p*-value = .0.808

Correlations are considered statistically significant at a Bonferroni corrected significance level of *p*-value < 0.017 and are highlighted with an asterisk. Only the pairs of RSNs surviving statistical comparison of the FBC—^a^autism spectrum disorder vs. typically developing controls; ^b^females vs. males; and ^c^females with autism spectrum disorder vs. typically developing female controls—were tested for correlation with ADI-R scores

## Discussion

The present study compares the between-RSNs FBC (within a set of 13 RSNs and applying ICA to rs-fMRI data) between individuals with ASD and TC, and between sexes, also exploring a potential modulation of the diagnosis effect by sex. Overall, our results may support the overarching disrupted connectivity hypothesis of ASD, involving the DM network (which showed abnormally increased connectivity with the executive control network in ASD vs. TC, and decreased connectivity with the cerebellum in males vs. females) and involving the HV, BG, visuospatial and language networks (only in females). In relation to specific RSNs, our findings may also support: (a) the executive dysfunction (Ozonoff et al., 1991); (b) the weak central coherence (Borup & Kølgaard, 2014; Frith, 1989); and (c) the empathizing-systemizing (Baron-Cohen, 2009) cognitive hypotheses of ASD, as discussed below.

### Default Mode Network Hyper-Connectivity with the Executive Control Network in ASD Compared to TC

We found a main effect of ASD diagnosis on the FBC between the DM and the REC networks, wherein individuals with ASD show higher connectivity than TC with a medium effect size (i.e. 71% of individuals with ASD were above the mean of the TC group’s connectivity [*Cohen’s d* = 0.56]). Abnormal connectivity involving the same area has been previously found, but in the form of *decreased* correlation in ASD between the cingulate gyrus network (the posterior part of which belongs to the DM network) and the bilateral executive control networks, in a smaller sample with male children and young adolescents (Bos et al., 2014). Additionally, a trend of decreased correlation in ASD (i.e. an effect that did not survive correction for multiple comparisons) between this pair of networks has been reported in a sample with mixed-sex children and adolescents (Olson et al., 2020). This study was developed in parallel to ours and used a sample partially from ABIDE I and II, i.e. that potentially overlaps with ours, and was of a size similar to ours herein. Moreover, although we do not replicate such findings, decreased between-RSN FBC in ASD has been reported between the DM and: (a) the salience networks [in a smaller sample with male adults (von dem Hagen et al., 2013)]; (b) the precuneus; and (c) the BG [using a smaller sample with mixed-sex children or adolescents, respectively, from the ABIDE I database, i.e. that potentially overlaps with ours; (Nomi & Uddin, 2015)] networks. The other two existing ASD between-RSNs FBC studies employing larger samples ((Oldehinkel et al., 2019)—one which consisted of children, adolescents and adults of both sexes (Cerliani et al., 2015), and another which consisted of male children, adolescents and adults from the ABIDE I database (i.e. potentially overlapping with our sample)—have not implicated the DM nor the executive control networks.

The DM network is responsible for social processes and has been extensively studied in ASD (Hull et al., 2017; Nair et al., 2020). It has also been predominantly found to have a decreased *within*-connectivity in ASD, which is associated with higher severity of ASD symptoms in adolescence and adulthood [for a complete review, see Hull et al., 2017; Nair et al., 2020)]. Furthermore, the DM network was recently reported to be less activated during performance in a mentalizing task, which in turn was associated with social communication deficits in adults with ASD (Hyatt et al., 2020). Furthermore, the executive control network is responsible for cognitive behavior adaptation to internal and external stimuli (Borup & Kølgaard, 2014) and has been found to be less activated in adolescents with ASD during the performance of cognitive control tasks (Solomon et al., 2009). Moreover, it has been hypothesized that the DM and the executive control networks are responsible for processing internal and external stimuli, respectively (Uddin & Menon, 2009). Therefore, disrupted connectivity between these two networks may underlie the impaired cognitive control ability usually seen in ASD (Solomon et al., 2008, 2009) by an inappropriate drive of ASD individuals to internally oriented processes (DM network) or to externally oriented processes (executive control network) (Menon & Uddin, 2010). As such, our results may also support the executive dysfunction cognitive hypothesis in ASD (Ozonoff et al., 1991).

### Default Mode Network Hyper-Connectivity with the Cerebellum Network in Males, Compared to Females

We found a main effect of sex on the FBC between the DM and the cerebellum network, with males showing higher connectivity than females with a medium effect size (i.e. 70% of males were above the mean of females’ connectivity [*Cohen’s d* = 0.51]). Decreased connectivity *within* the DM network has been previously found in TC males, compared to TC females (Olson et al., 2020; Stumme et al., 2020; Ypma et al., 2016). Additionally, although we do not replicate such findings, increased FBC in TC males compared to TC females has been reported between the DM and the executive control networks in older adults (Stumme et al., 2020). Regarding networks generally, there is growing evidence that females have an intensified network segregation (higher within-network connectivity), whereas males show an intensified network integration (higher between-network connectivity), and this has been suggested to be related to sex differences in behavior (i.e. usually males being better at systematizing tasks—motor and spatial cognitive tasks—and females better at empathizing tasks—emotion identification and nonverbal reasoning) (Satterthwaite et al., 2015; Stumme et al., 2020).

Furthermore, the cerebellum has been increasingly implicated in non-motor processes, such as social cognition and language (Sokolov et al., 2017). It has been hypothesized that, in addition to the sensorimotor prediction models, the cerebellum is important for the development of higher order cognitive prediction models, i.e. predicting (cognitive) output of higher order cognitive (e.g. language, social) functions and adapting to errors in this prediction (Popa & Ebner, 2019; Sokolov et al., 2017). The cerebellum is thought to shape the function of cortical brain areas responsible for these cognitive processes (e.g. frontal cortex—DM network) by being connected to them. Differences in the connectivity between these two networks might be linked to the difference in social skills between males and females. Furthermore, as expected, the males we studied showed higher mean social ADI-R score (20.1 ± 5.9) than females ([17.4 ± 5.6; although the two-sample *t*-test showed only a marginally significant difference between sexes (*p*-value = 0.058)].

### Visuospatial Network Hypo-Connectivity with the Language Network and High-Visual Network Hyper-Connectivity with the Basal Ganglia Network in ASD Females, Compared to TC Females

Although we did not find any effect of diagnosis being statistically significantly modulated by sex on FBC between any of the tested pairs of RSNs, we found a female-specific effect of diagnosis (i.e. that was not present in males) on the FBC between: (a) visuospatial and the language networks, with ASD females showing lower connectivity than TC females with a medium effect size (i.e. 75% of ASD females were below the mean of TC females’ connectivity [*Cohen’s d* = 0.68]); and (b) high-visual and BG networks, with ASD females showing *higher* connectivity than TC females with a medium effect size (i.e. 76% of ASD females were below the TC females’ connectivity mean [*Cohen’s d* = 0.71]). Interestingly, abnormal connectivity with the BG network has also been reported in the form of increased between-RSN FBC with the PV network in ASD, compared to TC, using a larger sample of only male children, adolescents and adults (Cerliani et al., 2015).

Visuospatial functions are reported to be intact or superior in ASD (Chabani & Hommel, 2014; Kana et al., 2013), whereas the opposite has consistently been found for language processing (Mody et al., 2013). Indeed, children with ASD, when compared to TC, have shown a preference for low level, perceptually oriented processing, increasing activation in occipito-parietal and ventral temporal areas (i.e. visuospatial network) and decreasing it in frontal and temporal language areas (i.e. language network) in a hybrid visuospatial-language processing task (Sahyoun et al., 2010). Moreover, the decreased activation in language areas is accompanied by a decrease in the integrity of the frontal–temporal fiber tracts (Sahyoun et al., 2010). Therefore, hypo-connectivity between these two networks is in line with the weak central coherence cognitive theory (Borup & Kølgaard, 2014; Frith, 1989). Furthermore, the fact that this difference is present in females but not in males may be in line with the empathizing-systemizing cognitive theory (Baron-Cohen, 2009): ASD females seem to be more similar to TC males (rely more on systemizing systems), than TC females (who rely more on empathizing systems).

The hyper-connectivity between cortical (HV network) and subcortical (BG network) networks in females with ASD may be also related to abnormal functional activation and volume in the BG. In particular, compared to TC, ASD individuals have shown (a) increased activation in the BG during a cognitive control task, in adults (Prat et al., 2016); and (b) decreased BG volumes, in children (Sussman et al., 2015), adolescents and adults (van Rooij et al., 2018)—previously found in ASD using mixed-sex samples. BG have a key role in balancing information flow to and from the cortex. Its disrupted connectivity is likely to be the culprit of a weak central coherence and cognitive executive dysfunction, which are primary cognitive hypotheses of ASD (Borup & Kølgaard, 2014; Vasa et al., 2016). Dysfunction in these structures has also been associated with over-responsivity to external stimuli in ASD, including in the visual sensory and sensorimotor domains in adults (Tavassoli et al., 2014) and repetitive, restrictive and stereotyped behaviors in male children, adolescents and adults (Schuetze et al., 2016).

### Limitations

Our study has a few limitations that need to be addressed. First, we included only subjects with a FIQ value > 70 and matched between sexes and diagnosis, which deems it more homogeneous and comparable to TC, but less representative of the real distribution of FIQ across populations. Second, the correlation analysis performed does not allow the direction of information flow between networks to be inferred—which would be an interesting exploration in a subsequent study. Third, our sample was selected from several sites, including rs-fMRI acquired either with eyes open or closed, and covered a wide age range (i.e. from children to adults). Although the groups of interest were balanced for site, eyes state at scan, and age and these extraneous variables were included as a covariate of no interest in our statistical analysis, the post hoc analysis of variance showed that: the interaction effect of ‘diagnosis by site’, ‘diagnosis by eyes state at scan’, ‘diagnosis by age’, ‘sex by site’, ‘sex by eyes state at scan’, and ‘sex by age’ on the functional brain connectivity of the pairs of resting-state networks that were shown to differ between (a) individuals with ASD and TC, (b) females and males, and (c) females with ASD and TC females. These were not statistically significant (*p* > 0.05; see Supplementary results and the Supplementary Table S14, in the Supplementary material). Fourth, we did not include total brain volume (TBV) as a covariate in our analysis. Although there is very little evidence of the impact of TBV on sex differences in resting state FBC (Eliot et al., 2020), we cannot fully exclude its potential confounding effect when analyzing sex effects in our sample.

## Conclusion

Our findings may support the overarching disrupted connectivity hypothesis of ASD (Vasa et al., 2016), involving the DM and the REC networks (using the whole sample) and the visuospatial and language networks, and high-visual and BG networks (only in females). Furthermore, our findings suggestively support the executive dysfunction, the weak central coherence and the empathizing-systemizing cognitive hypotheses of ASD. While further studies are needed to help understand current inconsistencies in the literature and further explore sex-specific brain functioning differences, between-RSN FBC is increasingly proving to be a promising tool for improving diagnostic biomarking and the etiological models of neurodevelopmental disorders, such as autism.

## Supplementary Information

Below is the link to the electronic supplementary material.

Supplementary file1 (DOCX 1725 kb)
