# Supplementary material for: Sex Differences in Functional Connectivity Between Resting State Brain Networks in Autism Spectrum Disorder
Source: J Autism Dev Disord. 2021 Jul 16;52(7):3088–101. doi: 10.1007/s10803-021-05191-6 (PMC9213274; doi:10.1007/s10803-021-05191-6)
Supplement: Supplementary file 1 — Supplementary file1 (DOCX 1725 kb) [file 10803_2021_5191_MOESM1_ESM.docx]

**Sex differences in functional connectivity between resting state brain networks in Autism Spectrum Disorder**

Vânia Tavares, Luís Afonso Fernandes, Marília Antunes, Hugo Ferreira, Diana Prata

**Supplementary material**

**Supplementary introduction**

In addition to the functional findings, structural neuroimaging has been extensively employed, producing robust findings of gray matter volume being increased in frontal (in both adults and children) (Eilam-Stock et al., 2016; Raznahan et al., 2013; van Rooij et al., 2018), parietal and middle temporal areas and decreased in the cerebellum and the cingulum in adults with ASD (Yang et al., 2016); with, moreover, the frontal increases associated with an increase in symptom severity in adults (Ecker et al., 2012; Grecucci et al., 2016). Decreased basal ganglia volumes (Ecker et al., 2012; Lai et al., 2015; Sussman et al., 2015; van Rooij et al., 2018) and both increased (Ecker et al., 2012; Yang et al., 2016) and decreased (van Rooij et al., 2018) amygdala volumes across lifespan have also been reported. Cortical thickness has also been reported to be increased in ASD (vs. TC, also across lifespan) in the frontal and superior temporal cortex, and precuneus and posterior cingulate cortices – an effect found to be male-specific (i.e. significant in males, but not in females) (Bedford et al., 2020). White matter-wise, it has generally been found a volume decrease and axonal disruption, especially in tracts connecting the anterior cingulate and frontal cortex (Radua et al., 2011; Zikopoulos et al., 2007) and in projection fibers within the cerebellum (D’Mello & Stoodley, 2015). In particular, disruption of frontal tracts has been shown to be increased in ASD males, when compared to TC, but not in ASD females, using a sample of adults. Furthermore, the more disrupted these tracts were, the more severe were the autism symptoms (Zeestraten et al., 2017).

**Supplementary methods**

Sample description

Data were downloaded from Autism Brain Imaging data exchange (ABIDE) repository (<http://fcon_1000.projects.nitrc.org/indi/abide/>) and included neuroimaging datasets from Georgetown University [GU, 2 females with Autism Spectrum Disorder (ASD-F), 2 males with ASD (ASD-M), 2 Typical female Controls (TC-F), and 2 Typical male Controls (TC-M)], Kennedy Krieger Institute (KKI, 6 ASD-F, 9 ASD-M, 9 TC-F, and 8 TC-M), New York University Langone Medical Center: Sample 1 (NYU_1, 10 ASD-F, 10 ASD-M, 10 TC-F, and 10 TC-M), San Diego State University (SDSU, 4 ASD-F, 4 ASD-M, 4 TC-F, and 4 TC-M), Stanford University: Sample 2 (SU_2, 1 ASD-F, 1 ASD-M, 1 TC-F, and 1 TC-M), University of California Davis (UCD, 3 ASD-F, 3 ASD-M, 3 TC-F, and 3 TC-M), University of Michigan: Sample 1 (UM_1, 6 ASD-F, 6 ASD-M, 6 TC-F, and 6 TC-M), University of Michigan: Sample 2 (UM_2, 1 ASD-F, 1 ASD-M, 1 TC-F, and 1 TC-M), and Yale Child Study Center (Yale, 7 ASD-F, 7 ASD-M, 7 TC-F, and 7 TC-M). Information regarding diagnostics and phenotypic assessment is detailed on the ABIDE website. Twenty-seven individual with ASD (15 females and 12 males) and 3 TC (1 female and 2 males) were on medication (detailed medication type is available in the phenotypic files on the ABDIE website). The IDs of the selected subjects are in **Supplementary Table S1**.

Sample selection

Inclusion of subjects in the final sample followed six main stages, described in detail below.

**Stage 1**: subjects must have information regarding age at scan, handedness, eye status at scan and full-scale intelligence quotient (FIQ), a FIQ higher than 70, and be right-handed. 450 subjects were excluded at this stage.

**Stage 2**: sites with no subjects belonging to one of the groups of interest (i.e. ASD-F, ASD-M, TC-F or TC-M) were excluded. 253 subjects from 7 sites were excluded at this stage (Barrow Neurological Institute; ETH Zürich; Katholieke Universiteit Leuven; Trinity Centre for Health Sciences; University of Leuven: Sample 1; NYU Langone Medical Center: Sample 2; and Social Brain Lab BCN NIC UMC Groningen and Netherlands Institute for Neurosciences).

**Stage 3**: subjects must have an anatomical T1-weighted image and a resting-state-functional Magnetic Resonance Imaging (rs-fMRI) with a full acquisition length 150 time points with at least 300 seconds and a near-full brain coverage; both T1-weighted and rs-fMRI must be free of excessive artifacts; and rs-fMRI must be successfully registered to T1-weighted image and MNI template. We, therefore, visually inspected 1) all anatomical raw images (examples of excluded images due to the presence of artifacts are in **Supplementary Figure S1**); and 2) all preprocessed mean and standard deviation functional images (examples of excluded images due to the presence of artifacts, not-full brain coverage, or bad registration to the anatomical and/or MNI template are in **Supplementary Figure S1**). 735 subjects were excluded at this stage.

**Stage 4**: sites with no subjects belonging to one of the groups of interest (i.e. ASD-F, ASD-M, TC-F or TC-M) were excluded. 133 subjects from 6 sites were excluded at this stage (Ludwig Maximilians, University Munich; California Institute of Technology; University of Miami; University of Utah School of Medicine; Stanford University: Sample 1; Carnegie Mellon University)

**Stage 5**: 57 subjects with excessive head motion, i.e. a maximum framewise displacement higher than 3 mm - the size of one functional voxel across the sample - were excluded. We ended this stage with 598 subjects (43 ASD-F, 172 ASD-M, 117 TC-F, 266 TC-M).

**Stage 6**: taking the ASD-F as a reference group (i.e. the group with the smallest size) we selected 43 subjects from the remaining three groups based on a one-to-one best match approach. For each subject in the ASD-F group, one subject from each of the other three groups (i.e. ASD-M, TC-F, TC-M) was selected based on the following criteria: a) belong to the same site; b) have the same eye status at scan; c) smallest sum of absolute differences in age, FIQ and head motion (i.e. mean framewise displacement). Diagnosis and sex groups were balanced for age, FIQ, and eye status across and within sites. Head motion was balanced between sexes, but not between diagnoses (*p*-value = .036) across sites, which was driven by the KKI site (*p*-value = .025). Therefore, we excluded 4 subjects (3 ASD-F and 1 TC-M) with mean framewise displacement in the lowest/highest 5^th^ percentile. The final sample was composed of 40 ASD-F, 43 ASD-M, 43 TC-F, and 42 TC-M.

Additional note on resting-state networks extraction, functional brain connectivity and statistical analyses

We have carefully inspected all the independent components and identified resting-state networks (RSNs) following standard guidelines described by (Kelly et al., 2010): a) minimal spatial overlap with vascular, ventricular and head-motion susceptible edge regions; b) a mean time course’s spectral power with a low-frequency range (0.01 ~ 0.1 Hz); and c) a spatial distribution overlap with RSNs masks, downloaded from the Functional Imaging in Neuropsychiatric Disorders (FIND) Lab at Stanford University (Shirer et al., 2012). Furthermore, independent component analysis (ICA) is a computational method able to separate a multivariate signal into statistically independent components (ICs). In this case, the multivariate signal is the blood oxygen level dependent (BOLD) signal acquired using resting state functional magnetic resonance imaging. The BOLD signal is a mixture of brain signal (i.e. grey or white matter) and non-brain signal (i.e. cerebrospinal fluid, motion, or vascular). ICA will separate these sources of brain and non-brain signal into ICs, which we can classify according to the criteria described above. In our analysis, we took advantage of this method and performed an intermediate step for data denoising using ICA-AROMA [ICA-based Automatic Removal Of Motion Artifacts; (Pruim et al., 2015)]. This is a data-driven method that identifies and regresses out head motion related independent components from the individual rs-fMRI data, i.e. the data denoising is performed for each subject individually. One clear advantage of this data denoising method is the reduction of the amount of noise to be inputted in the group ICA analysis. Herein, we have identified 13 ICs corresponding to RSNs and 7 ICs corresponding to non-RSNs, i.e. the ICs not resembling RSN networks as in (Shirer et al., 2012). We then discarded the non-RSNs ICs from further analyses and computed the functional connectivity between RSNs using only the 13 RSNs ICs. In sum, even though 1) we regressed head motion noise from individual rs-fMRI data and 2) the amount of head motion present in the data does not statically differ between groups (autism spectrum disorder *vs.* typical controls and females *vs.* males), in order to exclude any remaining effect of head motion (i.e. noise) present on the between resting-state functional brain connectivity (FBC), we included head motion as an extra regressor in our group statistical analyses.

Furthermore, pairwise differences between groups (i.e. ASD *vs.* TD, female *vs.* male) in the correlation coefficient (i.e. between pairs of resting-state networks) were measured using a general linear model with age, head motion (i.e. mean framewise displacement), eye status at scan, and site as covariates. The inclusion of the FIQ as a covariate did not change the reported statistically significant effects of diagnosis and sex on the between resting-state networks FBC post hoc t-tests.

Functional brain connectivity matrices

Let $\left\{ x_{ij}\left( t \right) \right\}, t=1,\ldots,n,n=147$, be the time series corresponding to the mean BOLD signal intensity in the $j-th$ resting-state network (RSN), $j=1,\ldots,13$, for the $i-th$ individual, $i=1,\ldots,168$. A FBC matrix $r^{\left( i \right)}$ was built for each subject computing the Pearson correlation coefficient for each possible pair $(j,k)$ of the 13 RSNs time courses (1),

$r_{jk}^{\left( i \right)}=\frac{\sum_{t=1}^{n} \left( x_{ij}\left( t \right)-\bar{x}_{ij} \right)\left( x_{ik}\left( t \right)-\bar{x}_{ik} \right)}{\sqrt{\sum_{t=1}^{n} \left( x_{ij}\left( t \right)-\bar{x}_{ij} \right)^{2}}\sqrt{\sum_{t=1}^{n} \left( x_{ik}\left( t \right)-{\bar{x}i}_{k} \right)^{2}}}, j,k=1,\ldots,13$ (1)

where $\bar{x}_{il}=\frac{1}{tn}\sum_{t=1}^{n} x_{il}\left( t \right)$ is the average of the mean intensity of the signal BOLD for the $i-th$ individual, $i=1,\ldots,168$, at the $l-th$ RSN, $l=1,\ldots,13$, over the $n=147$ instants of measurement. These 168 matrices were then normalized using Fisher’s z-transformation (2),

$z_{jk}^{\left( i \right)}=\frac{1}{2}\ln\left( \frac{1+r_{jk}^{\left( i \right)}}{1-r_{jk}^{\left( i \right)}} \right)$ (2)

Statistical analysis

A general linear model was used to measure the main effects of diagnosis and sex, the interaction effect of diagnosis by sex, and the pairwise differences between groups (diagnosis: ASD *vs* TD, sex: females *vs.* males) in the connectivity strength with age, head motion, eye status at scan and site as covariates (3),

$z_{jk}^{\left( i \right)}=\beta_{0}+\beta_{1}{diagnosis}^{(i)}+\beta_{2}{sex}^{(i)}+\beta_{3}{age}^{(i)}+\beta_{4}{head motion}^{(i)}+\beta_{5}{eye status at scan}^{(i)}+\beta_{6}{site}^{(i)}$ (3)

Effect size – *Cohen’s d*

The effect size of the group comparison (i.e. ASD *vs* TD, females *vs.* males) for each $(j,k)$ of RSNs mean time course pair, $d_{jk}$, was computed using *Cohen’s d* according to (4):

$d_{jk}=\frac{\bar{z}_{j,k,group1}-\bar{z}_{j,k,group2}}{\sqrt{\frac{\sigma_{j,k,group1}^{2}+\sigma_{j,k,group2}^{2}}{2}}}$, (4)

where $\bar{z}_{j,k,group1}$ and $\bar{z}_{j,k,group2}$ represent the mean z-score Pearson correlation coefficients of the $(j,k)$ pair of RSNs for the first and the second groups in the comparison. $\sigma_{j,k,group1}^{2}$ and $\sigma_{j,k,group2}^{2}$ represent the standard deviation of the z-score Pearson correlation coefficients of the $(j,k)$ pair of RSNs for the first and the second groups in the comparison.

**Supplementary results**

Site, age, and eye state at scan effects on between resting-state networks functional brain connectivity

The effects of diagnosis or sex by site, eye state at scan or age interaction on the FBC of pairs of RSNs was tested with a post hoc analysis of variance (ANOVA) for pairs shown to differ between a) individuals with ASD and TC (i.e. test the diagnosis by site, eye state at scan or age interaction effects on FBC of the ‘default mode – right executive control’ networks using the whole sample); b) females and males (i.e. test the sex by site, eye state at scan or age interaction effects on FBC of the ‘default mode – cerebellum’ networks interaction using the whole sample); and c) females with ASD and TC females (i.e. test the diagnosis by site, eye state at scan or age interaction effects on FBC of the ‘visuospatial – language’ and the ‘high visual – basal ganglia’ networks using only females). None of the tests were statistically significant (at a significance level of *p*-value < .05; see **Supplementary Table S14** for full statistics).

**References**

Bedford, S. A., Park, M. T. M., Devenyi, G. A., Tullo, S., Germann, J., Patel, R., Anagnostou, E., Baron-Cohen, S., Bullmore, E. T., Chura, L. R., Craig, M. C., Ecker, C., Floris, D. L., Holt, R. J., Lenroot, R., Lerch, J. P., Lombardo, M. V., Murphy, D. G. M., Raznahan, A., … Chakravarty, M. M. (2020). Large-scale analyses of the relationship between sex, age and intelligence quotient heterogeneity and cortical morphometry in autism spectrum disorder. *Molecular Psychiatry*, *25*(3), 614–628. https://doi.org/10.1038/s41380-019-0420-6

D’Mello, A. M., & Stoodley, C. J. (2015). Cerebro-cerebellar circuits in autism spectrum disorder. *Frontiers in Neuroscience*, *9*(NOV). https://doi.org/10.3389/fnins.2015.00408

Ecker, C., Suckling, J., Deoni, S. C., Lombardo, M. V., Bullmore, E. T., Baron-Cohen, S., Catani, M., Jezzard, P., Barnes, A., Bailey, A. J., Williams, S. C., Murphy, D. G. M., & Consortium, for the M. A. (2012). Brain Anatomy and Its Relationship to Behavior in Adults With Autism Spectrum Disorder. *Archives of General Psychiatry*, *69*(2), 195. https://doi.org/10.1001/archgenpsychiatry.2011.1251

Eilam-Stock, T., Wu, T., Spagna, A., Egan, L. J., & Fan, J. (2016). Neuroanatomical alterations in high-functioning adults with autism spectrum disorder. *Frontiers in Neuroscience*, *10*(JUN), 1–10. https://doi.org/10.3389/fnins.2016.00237

Grecucci, A., Rubicondo, D., Siugzdaite, R., Surian, L., & Job, R. (2016). Uncovering the social deficits in the autistic brain. A source-based morphometric study. *Frontiers in Neuroscience*, *10*(AUG), 1–8. https://doi.org/10.3389/fnins.2016.00388

Kelly, R. E., Alexopoulos, G. S., Wang, Z., Gunning, F. M., Murphy, C. F., Morimoto, S. S., Kanellopoulos, D., Jia, Z., Lim, K. O., & Hoptman, M. J. (2010). Visual inspection of independent components: defining a procedure for artifact removal from fMRI data. *Journal of Neuroscience Methods*, *189*(2), 233–245. https://doi.org/10.1016/j.jneumeth.2010.03.028

Lai, M. C., Lombardo, M. V., Ecker, C., Chakrabarti, B., Suckling, J., Bullmore, E. T., Happé, F., Murphy, D. G. M., & Baron-Cohen, S. (2015). Neuroanatomy of individual differences in language in adult males with autism. *Cerebral Cortex*, *25*(10), 3613–3628. https://doi.org/10.1093/cercor/bhu211

Pruim, R. H. R., Mennes, M., van Rooij, D., Llera, A., Buitelaar, J. K., & Beckmann, C. F. (2015). ICA-AROMA: A robust ICA-based strategy for removing motion artifacts from fMRI data. *NeuroImage*, *112*, 267–277. https://doi.org/10.1016/j.neuroimage.2015.02.064

Radua, J., Via, E., Catani, M., & Mataix-Cols, D. (2011). Voxel-based meta-analysis of regional white-matter volume differences in autism spectrum disorder versus healthy controls. *Psychological Medicine*, *41*(07), 1539–1550. https://doi.org/10.1017/S0033291710002187

Raznahan, A., Wallace, G. L., Antezana, L., Greenstein, D., Lenroot, R., Thurm, A., Gozzi, M., Spence, S., Martin, A., Swedo, S. E., & Giedd, J. N. (2013). Compared to what? Early brain overgrowth in autism and the perils of population norms. *Biological Psychiatry*, *74*(8), 563–575. https://doi.org/10.1016/j.biopsych.2013.03.022

Shirer, W. R., Ryali, S., Rykhlevskaia, E., Menon, V., & Greicius, M. D. (2012). Decoding Subject-Driven Cognitive States with Whole-Brain Connectivity Patterns. *Cerebral Cortex*, *22*(1), 158–165. https://doi.org/10.1093/cercor/bhr099

Sussman, D., Leung, R. C., Vogan, V. M., Lee, W., Trelle, S., Lin, S., Cassel, D. B., Chakravarty, M. M., Lerch, J. P., Anagnostou, E., & Taylor, M. J. (2015). The autism puzzle: Diffuse but not pervasive neuroanatomical abnormalities in children with ASD. *NeuroImage: Clinical*, *8*, 170–179. https://doi.org/10.1016/j.nicl.2015.04.008

van Rooij, D., Anagnostou, E., Arango, C., Auzias, G., Behrmann, M., Busatto, G. F., Calderoni, S., Daly, E., Deruelle, C., Di Martino, A., Dinstein, I., Duran, F. L. S., Durston, S., Ecker, C., Fair, D., Fedor, J., Fitzgerald, J., Freitag, C. M., Gallagher, L., … Buitelaar, J. K. (2018). Cortical and Subcortical Brain Morphometry Differences Between Patients With Autism Spectrum Disorder and Healthy Individuals Across the Lifespan: Results From the ENIGMA ASD Working Group. *American Journal of Psychiatry*, *175*(4), 359–369. https://doi.org/10.1176/appi.ajp.2017.17010100

Yang, X., Si, T., Gong, Q., Qiu, L., Jia, Z., Zhou, M., Zhao, Y., Hu, X., Wu, M., & Zhu, H. (2016). Brain gray matter alterations and associated demographic profiles in adults with autism spectrum disorder: A meta-analysis of voxel-based morphometry studies. *Australian & New Zealand Journal of Psychiatry*, *50*(8), 741–753. https://doi.org/10.1177/0004867415623858

Zeestraten, E. A., Gudbrandsen, M. C., Daly, E., de Schotten, M. T., Catani, M., Dell’Acqua, F., Lai, M.-C., Ruigrok, A. N. V, Lombardo, M. V., Chakrabarti, B., Baron-Cohen, S., Ecker, C., Murphy, D. G. M., & Craig, M. C. (2017). Sex differences in frontal lobe connectivity in adults with autism spectrum conditions. *Translational Psychiatry*, *7*(4), e1090–e1090. https://doi.org/10.1038/tp.2017.9

Zikopoulos, B., Barbas, H., Blatt, G., Chakravarti, A., Hof, P., Towns, L., Wagers, M., Wyatt, K., Meroni, P., Cetin, I., Derksen, R., Branch, W., Motta, M., Gordon, C., Ruiz-Irastorza, G., Spinillo, A., Friedman, D., Cimaz, R., Czeizel, A., & Piette, J. (2007). Parallel driving and modulatory pathways link the prefrontal cortex and thalamus. *PloS One*, *2*(9), e848. https://doi.org/10.1371/journal.pone.0000848

**Supplementary Table S1**- Selected subjects’ identifiers, diagnosis, sex and site from which their imaging data was selected from. Subjects excluded from the analysis due to head motion statistical differences between sexes are highlighted in grey. ASD: autism spectrum disorder; F: female; KKI: Kennedy Krieger Institute; NYU_1: New York University Langone Medical Center: Sample 1; SDSU: San Diego State University; SU_2: Stanford University: Sample 2; M: male; TC: typical controls; UCD: University of California Davis; UM_1: University of Michigan: Sample 1; UM_2: University of Michigan: Sample 2; Yale: Yale Child Study Center.

| **Identifier** | **Diagnosis** | **Sex** | **Site** | **Identifier** | **Diagnosis** | **Sex** | **Site** |
| --- | --- | --- | --- | --- | --- | --- | --- |
| **28761** | ASD | M | GU | **29337** | TC | F | KKI |
| **28779** | ASD | F | GU | **29338** | TC | M | KKI |
| **28780** | ASD | F | GU | **29344** | ASD | F | KKI |
| **28792** | TC | M | GU | **29355** | TC | M | KKI |
| **28797** | TC | M | GU | **29364** | TC | F | KKI |
| **28809** | ASD | M | GU | **29375** | ASD | F | KKI |
| **28814** | TC | F | GU | **29377** | TC | M | KKI |
| **28826** | TC | F | GU | **29381** | TC | M | KKI |
| **28853** | ASD | M | SDSU | **29389** | ASD | M | KKI |
| **28869** | ASD | F | SDSU | **29401** | ASD | M | KKI |
| **28871** | ASD | F | SDSU | **29409** | ASD | F | KKI |
| **28880** | TC | F | SDSU | **29411** | ASD | F | KKI |
| **28882** | TC | F | SDSU | **29412** | ASD | F | KKI |
| **28884** | ASD | M | SDSU | **29435** | ASD | M | KKI |
| **28895** | TC | M | SDSU | **29439** | TC | F | KKI |
| **28897** | ASD | M | SDSU | **29457** | TC | F | KKI |
| **28903** | ASD | F | SDSU | **29470** | TC | M | KKI |
| **28904** | TC | M | SDSU | **29474** | TC | M | KKI |
| **28907** | ASD | F | SDSU | **29477** | ASD | F | KKI |
| **28908** | ASD | M | SDSU | **29478** | TC | M | KKI |
| **29177** | ASD | F | NYU_1 | **29481** | ASD | M | KKI |
| **29184** | ASD | M | NYU_1 | **29998** | TC | F | UCD |
| **29198** | ASD | M | NYU_1 | **30001** | ASD | F | UCD |
| **29201** | ASD | M | NYU_1 | **30004** | TC | M | UCD |
| **29212** | ASD | F | NYU_1 | **30006** | TC | F | UCD |
| **29217** | ASD | F | NYU_1 | **30008** | TC | F | UCD |
| **29229** | TC | F | NYU_1 | **30009** | TC | M | UCD |
| **29238** | TC | M | NYU_1 | **30014** | ASD | M | UCD |
| **29242** | TC | F | NYU_1 | **30019** | ASD | M | UCD |
| **29247** | TC | M | NYU_1 | **30021** | ASD | F | UCD |
| **29286** | ASD | M | KKI | **30024** | TC | M | UCD |
| **29287** | ASD | F | KKI | **30026** | ASD | F | UCD |
| **29309** | TC | F | KKI | **30028** | ASD | M | UCD |
| **29334** | TC | F | KKI | **30172** | ASD | M | SU |
| **29336** | TC | F | KKI | **30185** | ASD | F | SU |

**Supplementary Table S1**-(cont.)

| **Identifier** | **Diagnosis** | **Sex** | **Site** | **Identifier** | **Diagnosis** | **Sex** | **Site** |
| --- | --- | --- | --- | --- | --- | --- | --- |
| **30192** | TC | M | SU | **50574** | TC | M | Yale |
| **30208** | TC | F | SU | **50576** | TC | F | Yale |
| **50195** | TC | M | SDSU | **50577** | TC | M | Yale |
| **50208** | TC | F | SDSU | **50604** | ASD | F | Yale |
| **50213** | TC | F | SDSU | **50609** | ASD | M | Yale |
| **50214** | TC | M | SDSU | **50611** | ASD | M | Yale |
| **50273** | ASD | M | UM_1 | **50612** | ASD | M | Yale |
| **50284** | ASD | F | UM_1 | **50614** | ASD | M | Yale |
| **50285** | ASD | F | UM_1 | **50616** | ASD | M | Yale |
| **50291** | ASD | M | UM_1 | **50619** | ASD | F | Yale |
| **50292** | ASD | M | UM_1 | **50620** | ASD | F | Yale |
| **50294** | ASD | M | UM_1 | **50621** | ASD | F | Yale |
| **50298** | ASD | M | UM_1 | **50622** | ASD | M | Yale |
| **50300** | ASD | F | UM_1 | **50623** | ASD | F | Yale |
| **50302** | ASD | F | UM_1 | **50624** | ASD | F | Yale |
| **50314** | ASD | M | UM_1 | **50627** | ASD | F | Yale |
| **50319** | ASD | F | UM_1 | **50628** | ASD | M | Yale |
| **50321** | ASD | F | UM_1 | **50788** | TC | F | KKI |
| **50329** | TC | M | UM_1 | **50795** | ASD | F | KKI |
| **50340** | TC | F | UM_1 | **50796** | ASD | F | KKI |
| **50343** | TC | F | UM_1 | **50797** | ASD | M | KKI |
| **50347** | TC | M | UM_1 | **50801** | ASD | M | KKI |
| **50353** | TC | F | UM_1 | **50810** | TC | F | KKI |
| **50354** | TC | F | UM_1 | **50813** | TC | M | KKI |
| **50357** | TC | F | UM_1 | **50818** | TC | M | KKI |
| **50358** | TC | M | UM_1 | **50823** | ASD | M | KKI |
| **50359** | TC | M | UM_1 | **50824** | ASD | M | KKI |
| **50369** | TC | F | UM_1 | **50953** | ASD | F | NYU_1 |
| **50370** | TC | M | UM_1 | **50954** | ASD | F | NYU_1 |
| **50377** | TC | M | UM_1 | **50957** | ASD | F | NYU_1 |
| **50390** | TC | M | UM_2 | **50959** | ASD | F | NYU_1 |
| **50399** | ASD | M | UM_2 | **50960** | ASD | F | NYU_1 |
| **50407** | ASD | F | UM_2 | **50961** | ASD | F | NYU_1 |
| **50414** | TC | F | UM_2 | **50962** | ASD | F | NYU_1 |
| **50551** | TC | M | Yale | **50965** | ASD | M | NYU_1 |
| **50554** | TC | M | Yale | **50966** | ASD | M | NYU_1 |
| **50555** | TC | F | Yale | **50974** | ASD | M | NYU_1 |
| **50557** | TC | F | Yale | **51001** | ASD | M | NYU_1 |
| **50558** | TC | F | Yale | **51018** | ASD | M | NYU_1 |
| **50561** | TC | M | Yale | **51023** | ASD | M | NYU_1 |
| **50565** | TC | F | Yale | **51025** | ASD | M | NYU_1 |
| **50568** | TC | M | Yale | **51045** | TC | F | NYU_1 |
| **50569** | TC | F | Yale | **51050** | TC | F | NYU_1 |
| **50571** | TC | M | Yale | **51051** | TC | F | NYU_1 |
| **50572** | TC | F | Yale | **51054** | TC | F | NYU_1 |

**Supplementary Table S1**-(cont.)

| **Identifier** | **Diagnosis** | **Sex** | **Site** | **Identifier** | **Diagnosis** | **Sex** | **Site** |
| --- | --- | --- | --- | --- | --- | --- | --- |
| **51055** | TC | F | NYU_1 | **51105** | TC | M | NYU_1 |
| **51056** | TC | F | NYU_1 | **51106** | TC | M | NYU_1 |
| **51060** | TC | F | NYU_1 | **51115** | TC | M | NYU_1 |
| **51062** | TC | F | NYU_1 | **51116** | TC | M | NYU_1 |
| **51068** | TC | M | NYU_1 | **51117** | TC | M | NYU_1 |
| **51077** | TC | M | NYU_1 | **51126** | TC | M | NYU_1 |

**Table S2**- Participants’ demographics by site. Group comparisons were made with a Mann-Whitney U-test for independent samples age at scan, FIQ, mean framewise displacement, ADI, and Social Responsiveness Scale and Chi-square test for eye state at scan. ADI-R: autism diagnostic interview-revised; ASD: autism spectrum disorder; C: closed eyes; F: female; FIQ: full scale intelligence quotient; M: male; O: open eyes; RRB: restrictive, repetitive, and stereotyped patterns of behavior. *Statistically significant at p-value < .05.

| **Georgetown University** | **ASD-F**  (n=2) | **ASD-M**  (n=2) | **TC-F**  (n=2) | **TC-M**  (n=2) | **Group comparison**  (*p*-value) |
| --- | --- | --- | --- | --- | --- |
| **Age^a^** (years) | 10.89 (0.0) | 11.7 (0.4)  [11.5, 12.0] | 9.2 (1.3)  [8.3, 10.1] | 12.3 (2.1)  [10.9, 13.8] | ASD vs. TD: .248  F vs. M: .083  ASD.F vs. ASD.M: .121  TC.F vs. TC.M: .121  ASD.F vs. TC.F: .121  ASD.M vs. TC.M: 1 |
| **FIQ^a^** | 109.0 (24.0)  [92, 126] | 111.5 (23.3)  [95, 128] | 110.0 (21.2)  [95, 125] | 111.5 (19.1)  [98, 125] | ASD vs. TD: .884  F vs. M: .559  ASD.F vs. ASD.M: .439  TC.F vs. TC.M: .683  ASD.F vs. TC.F: 1  ASD.M vs. TC.M: 1 |
| **ADI -R Social^a^** | 18.0 (4.2)  [15, 21] | 17.5 (6.4)  [13, 22] | - | - | ASD.F vs. ASD.M: 1 |
| **ADI-R Verbal^a^** | 13.0 (5.7)  [9, 17] | 15.5 (2.1)  [14, 17] | - | - | ASD.F vs. ASD.M: .683 |
| **ADI-R RRB^a^** | 3.5 (0.7)  [3, 4] | 5.5 (0.7)  [5, 6] | - | - | ASD.F vs. ASD.M: .121 |
| **Social Responsiveness Scale^a^** | 93.4 (29.9)  [17,137] | 93.1 (27.9)  [42, 155] | 19.7 (12.5)  [2, 54] | 22.4 (19.4)  [2, 85] | ASD vs. TD: .043*  F vs. M: .564  ASD.F vs. ASD.M: 1  TC.F vs. TC.M: .121  ASD.F vs. TC.F: .121  ASD.M vs. TC.M: .439 |
| **Mean framewise displacement** (mm) | 0.10 (0.12)  [0.93, 0.11] | 0.11 (0.06)  [0.07, 0.15] | 0.09 (0.01)  [0.09, 0.10] | 0.05 (0.01)  [0.05, 0.06] | ASD vs. TD: .149  F vs. M: .248  ASD.F vs. ASD.M: 1  TC.F vs. TC.M: .333  ASD.F vs. TC.F: .439  ASD.M vs. TC.M: .121 |

^a^Data format: mean (standard deviation); [minimum, maximum]. All subjects had their eyes open at scan.

**Table S2**- (cont.)

| **Kennedy Krieger Institute** | **ASD-F**  (n=6) | **ASD-M**  (n=9) | **TC-F**  (n=9) | **TC-M**  (n=8) | **Group comparison**  (*p*-value) |
| --- | --- | --- | --- | --- | --- |
| **Age^a^** (years) | 10.3 (1.4)  [8.1, 11.7] | 10.7 (1.2)  [8.5, 12.5] | 10.0 (1.5)  [8.1, 12.4] | 11.0 (1.1)  [9.2, 12.7] | ASD vs. TD: .865  F vs. M: .126  ASD.F vs. ASD.M: .480  TC.F vs. TC.M: .124  ASD.F vs. TC.F: .906  ASD.M vs. TC.M: .773 |
| **FIQ^a^** | 107.2 (11.8)  [92, 125] | 108.1 (12.8)  [90, 131] | 108.6 (12.9)  [89, 127] | 110.3 (12.8)  [97, 131] | ASD vs. TD: .677  F vs. M: .791  ASD.F vs. ASD.M: .906  TC.F vs. TC.M: .772  ASD.F vs. TC.F: .723  ASD.M vs. TC.M: .630 |
| **ADI -R Social^a^** | 17.0 (5.5)  [10, 24] | 22.4 (6.6)  [11, 29] | - | - | ASD.F vs. ASD.M: .067 |
| **ADI-R Verbal^a^** | 12.2 (5.1)  [4, 19] | 17.9 (4.9)  [12, 24] | - | - | ASD.F vs. ASD.M: .059 |
| **ADI-R RRB^a^** | 5.5 (1.9)  [3, 8] | 6.6 (2.4)  [3, 12] | - | - | ASD.F vs. ASD.M.: .463 |
| **Social Responsiveness Scale^a,b^** | 93.4 (29.9)  [17,137] | 93.1 (27.9)  [42, 155] | 19.7 (12.5)  [2, 54] | 22.4 (19.4)  [2, 85] | ASD vs. TD: <.001*  F vs. M: .926  ASD.F vs. ASD.M: 528  TC.F vs. TC.M: .567  ASD.F vs. TC.F: .004*  ASD.M vs. TC.M: .006* |
| **Mean framewise displacement** (mm) | 0.16 (0.05)  [0.11, 0.25] | 0.12 (0.07)  [0.06, 0.23] | 0.09 (0.05)  [0.05, 0.20] | 0.11 (0.07)  [0.04, 0.24] | ASD vs. TD: .033*  F vs. M: .850  ASD.F vs. ASD.M: .195  TC.F vs. TC.M: .810  ASD.F vs. TC.F: .010*  ASD.M vs. TC.M: .386 |

^a^Data format: mean (standard deviation); [minimum, maximum]. Information was not available for ^b^1 ASD-F, 4 ASD-M, 2 TC-F, and 2 TC-M participants. All subjects had their eyes open at scan.

**Table S2**- (cont.)

| **New York University Langone Medical Center – Sample 1** | **ASD-F**  (n=10) | **ASD-M**  (n=10) | **TC-F**  (n=10) | **TC-M**  (n=10) | **Group comparison**  (*p*-value) |
| --- | --- | --- | --- | --- | --- |
| **Age^a^** (years) | 20.4 (10.6)  [6.8, 38.8] | 13.4 (5.1)  [7.3, 20.6] | 16.7 (6.5)  [5.9, 27.8] | 18.9 (7.9)  [7.2, 31.8] | ASD vs. TD: .441  F vs. M: .473  ASD.F vs. ASD.M: .112  TC.F vs. TC.M: .406  ASD.F vs. TC.F: ..450  ASD.M vs. TC.M: .143 |
| **FIQ^a^** | 99.2 (19.8)  [74, 132] | 101.4 (17.5)  [75, 132] | 105.7 (16.1)  [80, 132] | 102.3 (17.0)  [81, 132] | ASD vs. TD: .440  F vs. M: .839  ASD.F vs. ASD.M: .940  TC.F vs. TC.M: .733  ASD.F vs. TC.F: .131  ASD.M vs. TC.M: .940 |
| **ADI -R Social^a,b^** | 16.9 (6.7)  [9, 25] | 19.2 (6.1)  [9, 27] | - | - | ASD.F vs. ASD.M: .476 |
| **ADI-R Verbal^a,b^** | 14.8 (5.5)  [8, 23] | 16.2 (4.5)  [10, 22] | - | - | ASD.F vs. ASD.M: .503 |
| **ADI-R RRB^a,b^** | 5.1 (1.7)  [2, 8] | 4.9 (3.3)  [1, 12] | - | - | ASD.F vs. ASD.M: .561 |
| **Social Responsiveness Scale^a,c^** | 93.4 (29.9)  [17,137] | 93.1 (27 .9)  [42, 155] | 19.7 (12.5)  [2, 54] | 22.4 (19.4)  [2, 85] | ASD vs. TD: <.001*  F vs. M: .528  ASD.F vs. ASD.M: .017*  TC.F vs. TC.M: .198  ASD.F vs. TC.F: .001*  ASD.M vs. TC.M: .001* |
| **Eye State** | 8 O/2 C | 8 O/2 C | 8 O/2 C | 8 O/2 C | ASD vs. TD: 1  F vs. M: 1  ASD.F vs. ASD.M: 1  TC.F vs. TC.M: 1  ASD.F vs. TC.F: 1  ASD.M vs. TC.M: 1 |
| **Mean framewise displacement** (mm) | 0.07 (0.04)  [0.04, 0.17] | 0.07 (0.02)  [0.04, 0.09] | 0.07 (0.03)  [0.04, 0.12] | 0.07 (0.04)  [0.04, 0.18] | ASD vs. TD: .607  F vs. M: .534  ASD.F vs. ASD.M: .940  TC.F vs. TC.M: .597  ASD.F vs. TC.F: .880  ASD.M vs. TC.M: .364 |

^a^Data format: mean (standard deviation); [minimum, maximum]. Information was not available for ^b^2 ASD-F; ^c^3 TC-F and 4 TC-M participants.

**Table S2**- (cont.)

| **San Diego State University** | **ASD-F**  (n=4) | **ASD-M**  (n=4) | **TC-F**  (n=4) | **TC-M**  (n=4) | **Group comparison**  (*p*-value) |
| --- | --- | --- | --- | --- | --- |
| **Age^a^** (years) | 12.8 (3.9)  [9.2, 18.0] | 13.5 (2.6)  [10.9, 16.6] | 12.3 (1.2)  [10.9, 13.8] | 14.1 (1.7)  [12.6, 16.2] | ASD vs. TD: .713  F vs. M: 227  ASD.F vs. ASD.M: .564  TC.F vs. TC.M: ..083  ASD.F vs. TC.F: .773  ASD.M vs. TC.M: .564 |
| **FIQ^a^** | 98.5 (16.5)  [88, 123] | 101.0 (14.8)  [91, 123] | 103.0 (16.5)  [88, 125] | 100.8 (15.0)  [91, 123] | ASD vs. TD: .711  F vs. M: 597  ASD.F vs. ASD.M: .381  TC.F vs. TC.M: ..885  ASD.F vs. TC.F: .663  ASD.M vs. TC.M: .883 |
| **ADI -R Social^a^** | 18.6 (1.0)  [18, 20] | 16.8 (3.9)  [13, 21] | - | - | ASD.F vs. ASD.M: .661 |
| **ADI-R Verbal^a^** | 14.0 (3.5)  [11, 19] | 15.3 (2.4)  [12, 17] | - | - | ASD.F vs. ASD.M: .559 |
| **ADI-R RRB^a^** | 6.8 (4.1)  [2, 12] | 5.5 (0.6)  [5, 6] | - | - | ASD.F vs. ASD.M: .372 |
| **Social Responsiveness Scale^a,b^** | 93.4 (29.9)  [17,137] | 93.1 (27.9)  [42, 155] | 19.7 (12.5)  [2, 54] | 22.4 (19.4)  [2, 85] | ASD vs. TD: .006*  F vs. M: .378  ASD.F vs. ASD.M: .110  TC.F vs. TC.M: 1  ASD.F vs. TC.F: .064  ASD.M vs. TC.M: .064 |
| **Mean framewise displacement** (mm) | 0.08 (0.04)  [0.04, 0.14] | 0.05 (0.01)  [0.04, 0.07] | 0.07 (0.05)  [0.03, 0.13] | 0.04 (0.01)  [0.03, 0.05] | ASD vs. TD: .294  F vs. M: 401  ASD.F vs. ASD.M: .386  TC.F vs. TC.M: .773  ASD.F vs. TC.F: .386  ASD.M vs. TC.M: .386 |

^a^Data format: mean (standard deviation); [minimum, maximum]. Information was not available for ^b^2 TC-F and 2 TC-M participants. All subjects had their eyes open at scan.

**Table S2**- (cont.)

| **Stanford University – Sample 2** | **ASD-F**  (n=1) | **ASD-M**  (n=1) | **TC-F**  (n=1) | **TC-M**  (n=1) | **Group comparison**  (*p*-value) |
| --- | --- | --- | --- | --- | --- |
| **Age^a^** (years) | 13.0 | 10.9 | 9.8 | 12.6 | ASD vs. TD: .439  F vs. M: 1  ASD.F vs. ASD.M: 1  TC.F vs. TC.M: .317  ASD.F vs. TC.F: .317  ASD.M vs. TC.M: .317 |
| **FIQ^a^**  113 | 113 | 115 | 117 | 117 | ASD vs. TD: .102  F vs. M: .683  ASD.F vs. ASD.M: 1  TC.F vs. TC.M: 1  ASD.F vs. TC.F: .317  ASD.M vs. TC.M: .317 |
| **Mean framewise displacement** (mm) | .037 | .101 | .121 | .067 | ASD vs. TD: .439  F vs. M: 1  ASD.F vs. ASD.M: 1  TC.F vs. TC.M: .317  ASD.F vs. TC.F: .317  ASD.M vs. TC.M: .317 |

^a^Data format: mean (standard deviation); [minimum, maximum]. Information regarding ADI -R Social, Verbal, and RRB, and Social Responsiveness Scale was not available for all participants. All subjects had their eyes closed at scan.

**Table S2**- (cont.)

| **University of California Davis** | **ASD-F**  (n=3) | **ASD-M**  (n=3) | **TC-F**  (n=3) | **TC-M**  (n=3) | **Group comparison**  (*p*-value) |
| --- | --- | --- | --- | --- | --- |
| **Age^a^** (years) | 15.7 (1.7)  [13.8, 16.8] | 14.9 (0.6)  [14.5, 15.6] | 14.7 (1.4)  [13.8, 16.3] | 15.9 (1.6)  [14.1, 17.2] | ASD vs. TD: .873  F vs. M: .575  ASD.F vs. ASD.M: .513  TC.F vs. TC.M: .184  ASD.F vs. TC.F: .513  ASD.M vs. TC.M: .513 |
| **FIQ^a^** | 100.3 (12.7)  [86, 110] | 101.7 (9.1)  [92, 110] | 113.7 (18.9)  [92, 127] | 107.3 (2.1)  [105, 109] | ASD vs. TD: .260  F vs. M: .687  ASD.F vs. ASD.M: 1  TC.F vs. TC.M: .513  ASD.F vs. TC.F: .275  ASD.M vs. TC.M: .513 |
| **Social Responsiveness Scale^a^** | 93.4 (29.9)  [17,137] | 93.1 (27.9)  [42, 155] | 19.7 (12.5)  [2, 54] | 22.4 (19.4)  [2, 85] | ASD vs. TD: .010*  F vs. M: .630  ASD.F vs. ASD.M: .507  TC.F vs. TC.M: .827  ASD.F vs. TC.F: .127  ASD.M vs. TC.M: .046* |
| **Mean framewise displacement** (mm) | 0.06 (0.01)  [0.06, 0.07] | 0.05 (0.02)  [0.04, 0.08] | 0.06 (0.03)  [0.04, 0.09] | 0.06 (0.02)  [0.03, 0.07] | ASD vs. TD: .749  F vs. M: .631  ASD.F vs. ASD.M: .700  TC.F vs. TC.M: .827  ASD.F vs. TC.F: .513  ASD.M vs. TC.M: .827 |

^a^Data format: mean (standard deviation); [minimum, maximum]. Information regarding ADI -R Social, Verbal, and RRB was not available for all participants. All subjects had their eyes closed at scan.

**Table S2**- (cont.)

| **University of Michigan: Sample 1** | **ASD-F**  (n=6) | **ASD-M**  (n=6) | **TC-F**  (n=6) | **TC-M**  (n=6) | **Group comparison**  (*p*-value) |
| --- | --- | --- | --- | --- | --- |
| **Age^a^** (years) | 12.9 (3.2)  [9.7, 18.0] | 14.1 (1.9)  [12.6, 16.8] | 13.6 (2.2)  [9.5, 16.1] | 12.9 (2.9)  [9.8, 17.1] | ASD vs. TD: .954  F vs. M: .862  ASD.F vs. ASD.M: .377  TC.F vs. TC.M: .631  ASD.F vs. TC.F: .631  ASD.M vs. TC.M: .377 |
| **FIQ^a^** | 103.7 (12.9)  [90, 126] | 104.7 (12.9)  [90, 126] | 102.6 (13.1)  [89, 125] | 102.3 (13.4)  [85, 122] | ASD vs. TD: .644  F vs. M: .795  ASD.F vs. ASD.M: .810  TC.F vs. TC.M: 1  ASD.F vs. TC.F: .631  ASD.M vs. TC.M: .631 |
| **ADI -R Social^a^** | 18.5 (6.7)  [8, 25] | 19.5 (6.0)  [9, 25] | - | - | ASD.F vs. ASD.M: .747 |
| **ADI-R Verbal^a^** | 13.0 (3.9)  [6, 17] | 14.7 (3.3)  [9, 18] | - | - | ASD.F vs. ASD.M: .374 |
| **ADI-R RRB^a^** | 4.8 (1.2)  [3, 6] | 5.5 (2.3)  [3, 9] | - | - | ASD.F vs. ASD.M: .503 |
| **Mean framewise displacement** (mm) | 0.07 (0.02)  [0.05, 0.10] | 0.09 (0.06)  [0.04, 0.21] | 0.09 (0.06)  [0.03, 0.18] | 0.08 (0.09)  [0.03, 0.25] | ASD vs. TD: .225  F vs. M: .729  ASD.F vs. ASD.M: 873  TC.F vs. TC.M: .337  ASD.F vs. TC.F: .522  ASD.M vs. TC.M: .337 |

^a^Data format: mean (standard deviation); [minimum, maximum]. Information regarding Social Responsiveness Scale was not available for all participants. All subjects had their eyes open at scan.

**Table S2**- (cont.)

| **University of Michigan: Sample 2** | **ASD-F**  (n=1) | **ASD-M**  (n=1) | **TC-F**  (n=1) | **TC-M**  (n=1) | **Group comparison**  (*p*-value) |
| --- | --- | --- | --- | --- | --- |
| **Age^a^** (years) | 16.6 | 17.4 | 13.8 | 14.5 | ASD vs. TD: .121  F vs. M: .439  ASD.F vs. ASD.M: 1  TC.F vs. TC.M: .317  ASD.F vs. TC.F: .317  ASD.M vs. TC.M: .317 |
| **FIQ^a^** | 105 | 105 | 109 | 104 | ASD vs. TD: 1  F vs. M: .121  ASD.F vs. ASD.M: 1  TC.F vs. TC.M: .317  ASD.F vs. TC.F: .317  ASD.M vs. TC.M: .317 |
| **ADI -R Social^a^** | 13 | 16 | - | - | ASD.F vs. ASD.M: .317 |
| **ADI-R Verbal^a^** | 19 | 15 | - | - | ASD.F vs. ASD.M: .317 |
| **ADI-R RRB^a^** | 4 | 2 | - | - | ASD.F vs. ASD.M: .317 |
| **Mean framewise displacement** (mm) | 0.04 | 0.08 | 0.05 | 0.06 | ASD vs. TD: 1  F vs. M: 121  ASD.F vs. ASD.M: 1  TC.F vs. TC.M: .317  ASD.F vs. TC.F: .317  ASD.M vs. TC.M: .317 |

^a^Data format: mean (standard deviation); [minimum, maximum]. Information regarding Social Responsiveness Scale was not available for all participants. All subjects had their eyes open at scan.

**Table S2**- (cont.)

| **Yale Child Study Center** | **ASD-F**  (n=7) | **ASD-M**  (n=7) | **TC-F**  (n=7) | **TC-M**  (n=7) | **Group comparison**  (*p*-value) |
| --- | --- | --- | --- | --- | --- |
| **Age^a^** (years) | 13.5 (2.6)  [9.5, 16.6] | 12.4 (2.9)  [8.9, 17.8] | 13.5 (2.8)  [8.4, 16.7] | 12.7 (2.6)  [8.7, 15.9] | ASD vs. TD: .927  F vs. M: .323  ASD.F vs. ASD.M: .442  TC.F vs. TC.M: .522  ASD.F vs. TC.F: .898  ASD.M vs. TC.M: .848 |
| **FIQ^a^** | 91.7 (10.4)  [83, 114] | 93.1 (18.2)  [72, 121] | 107.1 (15.0)  [89, 131] | 93.0 (11.0)  [73, 110] | ASD vs. TD: .073  F vs. M: .482  ASD.F vs. ASD.M: 1  TC.F vs. TC.M: .109  ASD.F vs. TC.F: .035*  ASD.M vs. TC.M: .654 |
| **ADI -R Social^a,b^** | 17.2 (7.5)  [7, 27] | 22.1 (6.1)  [13, 28] | - | - | ASD.F vs. ASD.M: .328 |
| **ADI-R Verbal^a,b^** | 14.6 (4.6)  [10, 20] | 18.0 (4.0)  [11, 23] | - | - | ASD.F vs. ASD.M: .158 |
| **ADI-R RRB^a,b^** | 4.6 (3.7)  [0, 10] | 5.3 (3.0)  [1, 10] | - | - | ASD.F vs. ASD.M: .683 |
| **Social Responsiveness Scale^a,c^** | 93.4 (29.9)  [17,137] | 93.1 (27.9)  [42, 155] | 19.7 (12.5)  [2, 54] | 22.4 (19.4)  [2, 85] | ASD vs. TD: <.001*  F vs. M: .981  ASD.F vs. ASD.M: .701  TC.F vs. TC.M: .153  ASD.F vs. TC.F: .003*  ASD.M vs. TC.M: .010* |
| **Mean framewise displacement** (mm) | 0.09 (0.04)  [0.04, 0.18] | 0.09 (0.02)  [0.05, 0.11] | 0.07 (0.03)  [0.05, 0.13] | 0.07 (0.05)  [0.03, 0.17] | ASD vs. TD: .073  F vs. M: .927  ASD.F vs. ASD.M: .565  TC.F vs. TC.M: .482  ASD.F vs. TC.F: .338  ASD.M vs. TC.M: .225 |

^a^Data format: mean (standard deviation); [minimum, maximum]. Information was not available for ^b^2 ASD-F; ^c^1 TC-M participants. All subjects had their eyes open at scan.

**Supplementary Table S3-** Description of each independent component content.

| **Independent component** | **Description** |
| --- | --- |
| 1 | primary visual network |
| 2 | excluded from the analysis |
| 3 | excluded from the analysis |
| 4 | default mode network |
| 5 | excluded from the analysis |
| 6 | auditory network |
| 7 | high visual network |
| 8 | visuospatial network |
| 9 | precuneus network |
| 10 | cerebellum network |
| 11 | anterior salience network |
| 12 | excluded from the analysis |
| 13 | excluded from the analysis |
| 14 | excluded from the analysis |
| 15 | right executive control network |
| 16 | left executive control network |
| 17 | basal ganglia network |
| 18 | excluded from the analysis |
| 19 | language network |
| 20 | sensorimotor network |

**Supplementary Table S4-** Averaged functional connectivity matrices with mean z-scored Pearson correlation coefficients for each resting-state network pair, for each analyzed group: autism spectrum disorder – females, autism spectrum disorder – males, typical controls – females and typical controls – males. AS: anterior salience network; A: auditory network; BG: basal ganglia network; C: cerebellum network; DM: default mode network; HV: high visual network; L: language network; LEC: left executive control network; P: precuneus network; PV: primary visual network; REC: right executive control network; SM: sensorimotor network; VS: visuospatial network.

| **Autism spectrum disorder - females** | | | | | | | | | | | | | |
| --- | --- | --- | --- | --- | --- | --- | --- | --- | --- | --- | --- | --- | --- |
|  | **PV** | **DM** | **A** | **HV** | **VS** | **P** | **C** | **AS** | **REC** | **LEC** | **BG** | **L** | **SM** |
| **PV** | 0.00 | -0.04 | 0.28 | 4.58 | -0.74 | 1.18 | 0.17 | -1.66 | -3.41 | -2.85 | 0.03 | -1.95 | 0.84 |
| **DM** | -0.24 | 0.00 | -2.15 | -0.47 | -3.00 | 0.79 | -2.43 | 1.06 | 0.27 | 0.93 | -1.28 | 1.12 | -0.31 |
| **A** | -0.37 | -2.36 | 0.00 | -2.37 | 1.91 | -1.73 | 0.30 | 0.50 | -1.51 | -1.90 | 6.23 | 1.23 | 2.59 |
| **HV** | 5.21 | -0.39 | -3.02 | 0.00 | 1.06 | 0.69 | 0.25 | -2.58 | -1.58 | -1.90 | -0.69 | -2.34 | -0.69 |
| **VS** | -1.33 | -2.54 | 2.20 | 0.83 | 0.00 | 2.82 | 0.59 | -2.83 | -0.22 | -0.02 | -1.60 | -3.88 | 0.12 |
| **P** | 1.80 | 0.57 | -1.36 | 1.45 | 3.03 | 0.00 | -0.88 | -1.41 | 0.58 | -1.09 | -1.80 | -3.01 | -2.45 |
| **C** | -0.18 | -1.31 | 0.15 | -0.06 | 0.33 | -0.78 | 0.00 | -0.98 | -0.97 | -1.39 | 0.34 | -0.98 | 0.04 |
| **AS** | -1.05 | 0.00 | 1.28 | -2.10 | -2.55 | -1.67 | -0.73 | 0.00 | -0.61 | -0.79 | 1.88 | 3.65 | -0.65 |
| **REC** | -2.92 | 0.63 | -1.33 | -1.33 | -0.50 | 0.53 | -0.82 | -1.63 | 0.00 | 2.03 | -0.74 | -0.12 | -1.48 |
| **LEC** | -2.99 | 0.15 | -0.63 | -1.29 | 1.04 | -1.55 | -1.14 | -1.43 | 1.39 | 0.00 | -0.76 | 0.56 | -1.00 |
| **BG** | -0.41 | -1.59 | 6.14 | -1.66 | -0.96 | -1.10 | 0.52 | 1.82 | -0.62 | 0.16 | 0.00 | 0.72 | 0.72 |
| **L** | -1.46 | 0.82 | 1.65 | -2.11 | -3.05 | -2.57 | -0.93 | 3.88 | -1.07 | -0.02 | 0.59 | 0.00 | 0.21 |
| **SM** | 0.27 | -0.26 | 2.76 | -1.22 | 0.67 | -1.99 | 0.22 | -0.54 | -0.92 | -0.99 | 0.93 | 0.48 | 0.00 |
| **Autism spectrum disorder - males** | | | | | | | | | | | | | |

| **Typical controls - females** | | | | | | | | | | | | | |
| --- | --- | --- | --- | --- | --- | --- | --- | --- | --- | --- | --- | --- | --- |
|  | **PV** | **DM** | **A** | **HV** | **VS** | **P** | **C** | **AS** | **REC** | **LEC** | **BG** | **L** | **SM** |
| **PV** | 0.00 | -0.04 | -0.24 | 4.28 | -1.33 | 1.94 | 0.47 | -1.58 | -2.56 | -2.76 | -0.34 | -1.42 | 0.28 |
| **DM** | -0.16 | 0.00 | -2.11 | -0.77 | -2.94 | 1.07 | -2.05 | 1.45 | -0.83 | -0.30 | -1.53 | 0.77 | -0.14 |
| **A** | -0.47 | -1.85 | 0.00 | -3.60 | 2.44 | -0.96 | 0.10 | 0.28 | -0.62 | -0.96 | 5.38 | 2.13 | 2.49 |
| **HV** | 5.04 | -0.83 | -3.09 | 0.00 | 0.73 | 0.80 | 0.00 | -2.41 | -1.25 | -1.18 | -2.06 | -2.41 | -1.19 |
| **VS** | -1.28 | -2.62 | 2.37 | 0.85 | 0.00 | 2.46 | 0.69 | -2.92 | 0.06 | 0.79 | -1.28 | -2.51 | 0.88 |
| **P** | 1.22 | 0.48 | -1.66 | 1.09 | 3.10 | 0.00 | -0.06 | -1.06 | 0.60 | -2.04 | -1.31 | -2.52 | -1.85 |
| **C** | 0.30 | -1.35 | 0.35 | -0.36 | 0.02 | -0.49 | 0.00 | -1.07 | -0.52 | -1.66 | 0.49 | -1.40 | 0.34 |
| **AS** | -1.06 | 0.69 | 0.67 | -2.25 | -2.76 | -1.92 | -0.55 | 0.00 | -1.28 | -1.35 | 2.08 | 3.58 | -0.55 |
| **REC** | -3.11 | -0.99 | -0.70 | -2.04 | -0.67 | 0.28 | -0.97 | -0.96 | 0.00 | 1.96 | -0.02 | -0.96 | -1.13 |
| **LEC** | -3.17 | -0.37 | -0.50 | -1.97 | 0.74 | -1.41 | -1.65 | -1.12 | 1.81 | 0.00 | 0.01 | 0.26 | -0.79 |
| **BG** | -0.12 | -1.59 | 5.52 | -1.20 | -1.47 | -1.73 | 1.36 | 1.59 | 0.01 | 0.12 | 0.00 | 0.92 | 0.62 |
| **L** | -1.89 | 0.76 | 2.07 | -2.97 | -2.82 | -2.88 | -1.46 | 4.19 | -0.28 | 0.84 | 0.54 | 0.00 | 0.63 |
| **SM** | 0.48 | 0.07 | 2.38 | -0.85 | 0.63 | -2.27 | -0.19 | 0.03 | -0.91 | -0.59 | 0.25 | 0.84 | 0.00 |
| **Typical controls - males** | | | | | | | | | | | | | |

**Supplementary Table S5-** F-statistic and uncorrected and FWER-corrected p-values for main the effect of diagnosis. Uncorrected p-values < .05 and FWER-corrected p-values < .05 are highlighted in grey. AS: anterior salience network; A: auditory network; BG: basal ganglia network; C: cerebellum network; DM: default mode network; HV: high visual network; L: language network; LEC: left executive control network; P: precuneus network; PV: primary visual network; REC: right executive control network; SM: sensorimotor network; VS: visuospatial network.

| ***F*-statistic** | | | | | | | | | | | | | |
| --- | --- | --- | --- | --- | --- | --- | --- | --- | --- | --- | --- | --- | --- |
|  | **PV** | **DM** | **A** | **HV** | **VS** | **P** | **C** | **AS** | **REC** | **LEC** | **BG** | **L** | **SM** |
| **PV** | 0.00 | 0.00 | 1.31 | 0.01 | 0.42 | 0.70 | 1.65 | 0.08 | 1.62 | 0.00 | 0.00 | 0.02 | 0.54 |
| **DM** |  |  | 0.69 | 0.51 | 0.10 | 0.12 | 0.92 | 1.98 | 12.12 | 4.84 | 0.09 | 0.23 | 0.61 |
| **A** |  |  |  | 3.24 | 0.86 | 0.06 | 0.09 | 1.08 | 4.41 | 2.05 | 5.60 | 6.30 | 0.39 |
| **HV** |  |  |  |  | 0.58 | 0.02 | 0.51 | 0.04 | 0.99 | 0.07 | 2.11 | 1.66 | 0.13 |
| **VS** |  |  |  |  |  | 0.43 | 0.15 | 0.03 | 0.01 | 0.63 | 0.21 | 9.75 | 0.88 |
| **P** |  |  |  |  |  | 0.00 | 2.30 | 0.01 | 0.02 | 1.05 | 0.31 | 0.07 | 0.40 |
| **C** |  |  |  |  |  |  |  | 0.00 | 0.16 | 1.80 | 1.58 | 2.65 | 0.16 |
| **AS** |  |  |  |  |  |  |  | 000 | 0.01 | 0.08 | 0.03 | 0.00 | 2.67 |
| **REC** |  |  |  |  |  |  |  |  | 0 | 0.68 | 3.75 | 0.13 | 0.55 |
| **LEC** |  |  |  |  |  |  |  |  |  |  | 0.92 | 0.58 | 1.49 |
| **BG** |  |  |  |  |  |  |  |  |  |  |  | 0.24 | 1.32 |
| **L** |  |  |  |  |  |  |  |  |  |  |  |  | 2.60 |
| **SM** |  |  |  |  |  |  |  |  |  |  |  |  | .00 |
|  | | | | | | | | | | | | | |

| **Uncorrected *p*-value** | | | | | | | | | | | | | |
| --- | --- | --- | --- | --- | --- | --- | --- | --- | --- | --- | --- | --- | --- |
|  | **PV** | **DM** | **A** | **HV** | **VS** | **P** | **C** | **AS** | **REC** | **LEC** | **BG** | **L** | **SM** |
| **PV** | 1 | .957 | .255 | .906 | .518 | .403 | .197 | .783 | .206 | .963 | .950 | .888 | .468 |
| **DM** | 1 | 1 | .411 | .472 | .751 | .726 | .336 | .159 | .001 | .030 | .767 | .637 | .433 |
| **A** | 1 | 1 | 1 | .074 | .352 | .801 | .764 | .303 | .035 | .155 | .020 | .013 | .535 |
| **HV** | 1 | 1 | .990 | 1 | .449 | .900 | .477 | .841 | .315 | .787 | .146 | .200 | .725 |
| **VS** | 1 | 1 | 1 | 1 | 1 | .517 | .703 | .860 | .927 | .425 | .646 | .002 | .351 |
| **P** | 1 | 1 | 1 | 1 | 1 | 1 | .131 | .933 | .874 | .312 | .580 | .785 | .523 |
| **C** | 1 | 1 | 1 | 1 | 1 | 1 | 1 | .943 | .688 | .183 | .208 | .108 | .697 |
| **AS** | 1 | 1 | 1 | 1 | 1 | 1 | 1 | 1 | .925 | .775 | .867 | .977 | .105 |
| **REC** | 1 | .049 | .908 | 1 | 1 | 1 | 1 | 1 | 1 | .415 | .056 | .721 | .460 |
| **LEC** | 1 | .850 | 1 | 1 | 1 | 1 | 1 | 1 | 1 | 1 | .339 | .453 | .227 |
| **BG** | 1 | 1 | .720 | 1 | 1 | 1 | 1 | 1 | 0.969 | 1 | 1 | .627 | .259 |
| **L** | 1 | 1 | .588 | 1 | 0.143 | 1 | 0.999 | 1 | 1 | 1 | 1 | 1 | .110 |
| **SM** | 1 | 1 | 1 | 1 | 1 | 1 | 1 | 0.999 | 1 | 1 | 1 | .999 | 1 |
| **FWER-corrected *p*-value** | | | | | | | | | | | | | |

**Supplementary Table S6-** F-statistic and uncorrected and FWER-corrected p-values for the main effect of sex. Uncorrected p-values < .05 and FWER-corrected p-values < .05 are highlighted in grey. AS: anterior salience network; A: auditory network; BG: basal ganglia network; C: cerebellum network; DM: default mode network; HV: high visual network; L: language network; LEC: left executive control network; P: precuneus network; PV: primary visual network; REC: right executive control network; SM: sensorimotor network; VS: visuospatial network.

| ***F*-statistic** | | | | | | | | | | | | | |
| --- | --- | --- | --- | --- | --- | --- | --- | --- | --- | --- | --- | --- | --- |
|  | **PV** | **DM** | **A** | **HV** | **VS** | **P** | **C** | **AS** | **REC** | **LEC** | **BG** | **L** | **SM** |
| **PV** |  | 0.36 | 1.85 | 4.01 | 0.81 | 0.00 | 0.83 | 3.83 | 0.00 | 0.94 | 0.17 | 0.00 | 0.51 |
| **DM** |  |  | 0.01 | 0.04 | 2.24 | 1.65 | 12.22 | 6.81 | 0.13 | 1.65 | 0.45 | 0.20 | 0.19 |
| **A** |  |  |  | 0.03 | 0.06 | 0.29 | 0.00 | 4.07 | 0.01 | 8.73 | 0.00 | 0.36 | 0.00 |
| **HV** |  |  |  |  | 0.11 | 2.04 | 1.04 | 0.94 | 0.78 | 0.06 | 0.05 | 0.17 | 0.14 |
| **VS** |  |  |  |  |  | 1.57 | 2.45 | 0.63 | 2.34 | 2.53 | 0.47 | 0.78 | 0.17 |
| **P** |  |  |  |  |  |  | 0.34 | 3.02 | 0.26 | 0.08 | 0.16 | 0.01 | 0.01 |
| **C** |  |  |  |  |  |  |  | 1.41 | 0.32 | 0.40 | 2.49 | 0.01 | 0.66 |
| **AS** |  |  |  |  |  |  |  |  | 0.94 | 0.20 | 0.76 | 1.36 | 1.91 |
| **REC** |  |  |  |  |  |  |  |  |  | 1.05 | 0.02 | 0.17 | 2.04 |
| **LEC** |  |  |  |  |  |  |  |  |  |  | 3.01 | 0.01 | 0.18 |
| **BG** |  |  |  |  |  |  |  |  |  |  |  | 0.49 | 0.09 |
| **L** | 0.00 |  |  |  |  |  |  |  |  |  |  |  | 1.02 |
| **SM** | 0.51 | 0.19 | 0.00 |  |  |  |  |  |  | 0.18 | 0.09 |  |  |
|  | | | | | | | | | | | | | |

| **Uncorrected *p*-value** | | | | | | | | | | | | | |
| --- | --- | --- | --- | --- | --- | --- | --- | --- | --- | --- | --- | --- | --- |
|  | **PV** | **DM** | **A** | **HV** | **VS** | **P** | **C** | **AS** | **REC** | **LEC** | **BG** | **L** | **SM** |
| **PV** | 1 | .546 | .174 | .047 | .369 | .990 | .365 | .050 | .978 | .329 | .681 | .983 | .480 |
| **DM** | 1 | 1 | .927 | .851 | .132 | .202 | .001 | .010 | .718 | .201 | .510 | .652 | .657 |
| **A** | 1 | 1 | 1 | .867 | .802 | .591 | .993 | .046 | .914 | .004 | .978 | .550 | .952 |
| **HV** | .953 | 1 | 1 | 1 | .739 | .158 | .311 | .335 | .374 | .811 | .832 | .685 | .715 |
| **VS** | 1 | 1 | 1 | 1 | 1 | .211 | .122 | .428 | .128 | .117 | .487 | .374 | .682 |
| **P** | 1 | 1 | 1 | 1 | 1 | 1 | .557 | .085 | .613 | .767 | .688 | .903 | .903 |
| **C** | 1 | .046 | 1 | 1 | 1 | 1 | 1 | .233 | .569 | .521 | .118 | .934 | .416 |
| **AS** | .966 | .498 | .947 | 1 | 1 | .996 | 1 | 1 | .332 | .658 | .392 | .245 | .165 |
| **REC** | 1 | 1 | 1 | 1 | 1 | 1 | 1 | 1 | 1 | .310 | .880 | .682 | .151 |
| **LEC** | 1 | 1 | .233 | 1 | .999 | 1 | 1 | 1 | 1 | 1 | .082 | .929 | .667 |
| **BG** | 1 | 1 | 1 | 1 | 1 | 1 | .999 | 1 | 1 | .996 | 1 | .490 | .764 |
| **L** | 1 | 1 | 1 | 1 | 1 | 1 | 1 | 1 | 1 | 1 | 1 | 1 | .318 |
| **SM** | 1 | 1 | 1 | 1 | 1 | 1 | 1 | 1 | 1 | 1 | 1 | 1 | 1 |
| **FWER-corrected *p*-value** | | | | | | | | | | | | | |

**Supplementary Table S7-** F-statistic and uncorrected and FWER-corrected p-values for the interaction effect diagnosis by sex. Uncorrected p-values < .05 and FWER-corrected p-values < .05 are highlighted in grey. AS: anterior salience network; A: auditory network; BG: basal ganglia network; C: cerebellum network; DM: default mode network; HV: high visual network; L: language network; LEC: left executive control network; P: precuneus network; PV: primary visual network; REC: right executive control network; SM: sensorimotor network; VS: visuospatial network.

| ***F*-statistic** | | | | | | | | | | | | | |
| --- | --- | --- | --- | --- | --- | --- | --- | --- | --- | --- | --- | --- | --- |
|  | **PV** | **DM** | **A** | **HV** | **VS** | **P** | **C** | **AS** | **REC** | **LEC** | **BG** | **L** | **SM** |
| **PV** | 0.00 | 0.03 | 0.70 | 0.01 | 0.99 | 5.38 | 0.30 | 0.00 | 4.50 | 0.03 | 1.70 | 2.05 | 2.13 |
| **DM** | 0.03 | 0.00 | 0.48 | 0.12 | 0.05 | 0.32 | 0.73 | 0.00 | 0.70 | 1.79 | 0.34 | 0.53 | 0.08 |
| **A** | 0.70 | 0.48 | 0.00 | 3.90 | 0.18 | 3.52 | 0.49 | 0.13 | 0.12 | 1.61 | 0.23 | 0.49 | 0.11 |
| **HV** | 0.01 | 0.12 | 3.90 | 0.00 | 0.56 | 0.53 | 0.00 | 0.39 | 3.04 | 4.69 | 11.11 | 1.44 | 3.37 |
| **VS** | 0.99 | 0.05 | 0.18 | 0.56 | 0.00 | 0.12 | 0.54 | 0.01 | 0.62 | 2.70 | 1.92 | 3.15 | 1.06 |
| **P** | 5.38 | 0.32 | 3.52 | 0.53 | 0.12 | 0.00 | 1.01 | 0.47 | 0.08 | 4.67 | 3.61 | 1.40 | 2.45 |
| **C** | 0.30 | 0.73 | 0.49 | 0.00 | 0.54 | 1.01 | 0.00 | 0.31 | 1.58 | 0.95 | 0.80 | 0.04 | 1.63 |
| **AS** | 0.00 | 0.00 | 0.13 | 0.39 | 0.01 | 0.47 | 0.31 | 0.00 | 4.46 | 1.33 | 0.29 | 0.34 | 0.66 |
| **REC** | 4.50 | 0.70 | 0.12 | 3.04 | 0.62 | 0.08 | 1.58 | 4.46 | 0.00 | 0.37 | 0.00 | 5.73 | 0.25 |
| **LEC** | 0.03 | 1.79 | 1.61 | 4.69 | 2.70 | 4.67 | 0.95 | 1.33 | 0.37 | 0.00 | 1.96 | 2.33 | 0.21 |
| **BG** | 1.70 | 0.34 | 0.23 | 11.11 | 1.92 | 3.61 | 0.80 | 0.29 | 0.00 | 1.96 | 0.00 | 0.09 | 0.78 |
| **L** | 2.05 | 0.53 | 0.49 | 1.44 | 3.15 | 1.40 | 0.04 | 0.34 | 5.73 | 2.33 | 0.09 | 0.00 | 0.19 |
| **SM** | 2.13 | 0.08 | 0.11 | 3.37 | 1.06 | 2.45 | 1.63 | 0.66 | 0.25 | 0.21 | 0.78 | 0.19 | 0.00 |
|  | | | | | | | | | | | | | |

| **Uncorrected *p*-value** | | | | | | | | | | | | | |
| --- | --- | --- | --- | --- | --- | --- | --- | --- | --- | --- | --- | --- | --- |
|  | **PV** | **DM** | **A** | **HV** | **VS** | **P** | **C** | **AS** | **REC** | **LEC** | **BG** | **L** | **SM** |
| **PV** | 1 | .861 | .409 | .905 | .319 | .021 | .585 | .992 | .037 | .854 | .196 | .157 | .146 |
| **DM** | 1 | 1 | .486 | .731 | .821 | .574 | .399 | .990 | .408 | .184 | .557 | .470 | .789 |
| **A** | 1 | 1 | 1 | .050 | .676 | .061 | .482 | .722 | .726 | .210 | .634 | .488 | .738 |
| **HV** | 1 | 1 | .964 | 1 | .450 | .462 | .963 | .528 | .084 | .030 | .001 | .229 | .067 |
| **VS** | 1 | 1 | 1 | 1 | 1 | .720 | .465 | .910 | .427 | .101 | .165 | .079 | .307 |
| **P** | .769 | 1 | .983 | 1 | 1 | 1 | .321 | .495 | .769 | .032 | .057 | .240 | .119 |
| **C** | 1 | 1 | 1 | 1 | 1 | 1 | 1 | .585 | .212 | .338 | .377 | .841 | .210 |
| **AS** | 1 | 1 | 1 | 1 | 1 | 1 | 1 | 1 | .037 | .253 | .597 | .567 | .420 |
| **REC** | .909 | 1 | 1 | .995 | 1 | 1 | 1 | .914 | 1 | .548 | .947 | .018 | .622 |
| **LEC** | 1 | 1 | 1 | .884 | .998 | .887 | 1 | 1 | 1 | 1 | .168 | .130 | .646 |
| **BG** | 1 | 1 | 1 | .077 | 1 | .980 | 1 | 1 | 1 | 1 | 1 | .764 | .377 |
| **L** | 1 | 1 | 1 | 1 | .993 | 1 | 1 | 1 | .700 | 1 | 1 | 1 | .663 |
| **SM** | 1 | 1 | 1 | .988 | 1 | .999 | 1 | 1 | 1 | 1 | 1 | 1 | 1 |
| **FWER-corrected *p*-value** | | | | | | | | | | | | | |

**Supplementary Table S8-** t-statistic and effect size (Cohen’s d) and uncorrected and FWER-corrected p-values for autism spectrum disorder and typical controls group comparison. Uncorrected p-values < .05 and FWER-corrected p-values < .05 are highlighted in grey. AS: anterior salience network; A: auditory network; BG: basal ganglia network; C: cerebellum network; DM: default mode network; HV: high visual network; L: language network; LEC: left executive control network; P: precuneus network; PV: primary visual network; REC: right executive control network; SM: sensorimotor network; VS: visuospatial network.

| **Effect size** (*Cohen’s d*) | | | | | | | | | | | | | |
| --- | --- | --- | --- | --- | --- | --- | --- | --- | --- | --- | --- | --- | --- |
|  | **PV** | **DM** | **A** | **HV** | **VS** | **P** | **C** | **AS** | **REC** | **LEC** | **BG** | **L** | **SM** |
| **PV** | 0.00 | 0.03 | 0.13 | 0.09 | 0.14 | 0.04 | 0.20 | 0.01 | 0.17 | 0.02 | 0.02 | 0.03 | 0.10 |
| **DM** | 0.06 | 0.00 | 0.12 | 0.18 | 0.01 | 0.05 | 0.08 | 0.25 | 0.56 | 0.39 | 0.06 | 0.08 | 0.13 |
| **A** | 1.14 | 0.83 | 0.00 | 0.27 | 0.18 | 0.11 | 0.00 | 0.22 | 0.37 | 0.26 | 0.32 | 0.30 | 0.13 |
| **HV** | 0.12 | 0.71 | 1.80 | 0.00 | 0.07 | 0.06 | 0.13 | 0.00 | 0.09 | 0.01 | 0.23 | 0.20 | 0.03 |
| **VS** | 0.65 | 0.31 | 0.93 | 0.76 | 0.00 | 0.07 | 0.05 | 0.08 | 0.03 | 0.11 | 0.05 | 0.38 | 0.16 |
| **P** | 0.84 | 0.35 | 0.25 | 0.13 | 0.65 | 0.00 | 0.27 | 0.03 | 0.05 | 0.20 | 0.04 | 0.04 | 0.09 |
| **C** | 1.28 | 0.96 | 0.30 | 0.72 | 0.39 | 1.52 | 0.00 | 0.02 | 0.08 | 0.23 | 0.23 | 0.28 | 0.03 |
| **AS** | 0.27 | 1.41 | 1.04 | 0.20 | 0.18 | 0.08 | 0.07 | 0.00 | 0.01 | 0.05 | 0.01 | 0.05 | 0.20 |
| **REC** | 1.27 | 3.48 | 2.10 | 1.00 | 0.09 | 0.16 | 0.40 | 0.10 | 0.00 | 0.08 | 0.38 | 0.00 | 0.09 |
| **LEC** | 0.05 | 2.20 | 1.43 | 0.27 | 0.79 | 1.02 | 1.34 | 0.28 | 0.82 | 0.00 | 0.18 | 0.12 | 0.17 |
| **BG** | 0.06 | 0.30 | 2.37 | 1.45 | 0.46 | 0.56 | 1.26 | 0.17 | 1.94 | 0.96 | 0.00 | 0.04 | 0.20 |
| **L** | 0.14 | 0.47 | 2.51 | 1.29 | 3.12 | 0.26 | 1.63 | 0.03 | 0.36 | 0.76 | 0.49 | 0.00 | 0.22 |
| **SM** | 0.74 | 0.78 | 0.62 | 0.36 | 0.94 | 0.63 | 0.40 | 1.63 | 0.74 | 1.22 | 1.15 | 1.61 | 0.00 |
| ***t*-statistic** | | | | | | | | | | | | | |

| **Uncorrected *p*-value** (autism spectrum disorder > typical controls) | | | | | | | | | | | | | |
| --- | --- | --- | --- | --- | --- | --- | --- | --- | --- | --- | --- | --- | --- |
|  | **PV** | **DM** | **A** | **HV** | **VS** | **P** | **C** | **AS** | **REC** | **LEC** | **BG** | **L** | **SM** |
| **PV** | 1 | .479 | .128 | .451 | .260 | .796 | .904 | .392 | .895 | .480 | .476 | .445 | .231 |
| **DM** | 1 | 1 | .798 | .235 | .620 | .633 | .834 | .921 | .001 | .015 | .381 | .316 | .780 |
| **A** | 1 | 1 | 1 | .036 | .825 | .601 | .384 | .149 | .982 | .923 | .009 | .993 | .269 |
| **HV** | 1 | 1 | .930 | 1 | .225 | .447 | .238 | .422 | .160 | .605 | .072 | .100 | .361 |
| **VS** | 1 | 1 | 1 | 1 | 1 | .260 | .343 | .576 | .536 | .786 | .327 | .999 | .829 |
| **P** | 1 | 1 | 1 | 1 | 1 | 1 | .938 | .530 | .442 | .150 | .289 | .600 | .734 |
| **C** | 1 | 1 | 1 | 1 | 1 | 1 | 1 | .472 | .658 | .095 | .895 | .052 | .350 |
| **AS** | 1 | 1 | 1 | 1 | 1 | 1 | 1 | 1 | .538 | .385 | .562 | .489 | .948 |
| **REC** | 1 | .025 | 1 | 1 | 1 | 1 | 1 | 1 | 1 | .792 | .972 | .359 | .771 |
| **LEC** | 1 | .653 | 1 | 1 | 1 | 1 | .999 | 1 | 1 | 1 | .832 | .776 | .889 |
| **BG** | 1 | 1 | .507 | .996 | 1 | 1 | 1 | 1 | 1 | 1 | 1 | .687 | .123 |
| **L** | 1 | 1 | 1 | 1 | 1 | 1 | .978 | 1 | 1 | 1 | 1 | 1 | .943 |
| **SM** | 1 | 1 | 1 | 1 | 1 | 1 | 1 | 1 | 1 | 1 | 1 | 1 | 1 |
| **FWER-corrected *p*-value** (autism spectrum disorder > typical controls) | | | | | | | | | | | | | |

**Supplementary Table S8-** (cont.)

| **Uncorrected *p*-value** (typical controls > autism spectrum disorder) | | | | | | | | | | | | | |
| --- | --- | --- | --- | --- | --- | --- | --- | --- | --- | --- | --- | --- | --- |
|  | **PV** | **DM** | **A** | **HV** | **VS** | **P** | **C** | **AS** | **REC** | **LEC** | **BG** | **L** | **SM** |
| **PV** | 1 | .525 | .871 | .550 | .736 | .202 | .099 | .612 | .101 | .518 | .521 | .554 | .771 |
| **DM** | 1 | 1 | .199 | .761 | .373 | .365 | .169 | .081 | 1 | .986 | .618 | .682 | .216 |
| **A** | 1 | 1 | 1 | .962 | .180 | .398 | .625 | .844 | .021 | .075 | .990 | .007 | .736 |
| **HV** | 1 | 1 | 1 | 1 | .778 | .549 | .764 | .585 | .840 | .400 | .927 | .901 | .640 |
| **VS** | 1 | 1 | 1 | 1 | 1 | .742 | .650 | .428 | .463 | .212 | .679 | .001 | .174 |
| **P** | 1 | 1 | 1 | 1 | 1 | 1 | .064 | .472 | .559 | .850 | .714 | .396 | .259 |
| **C** | 1 | 1 | 1 | 1 | 1 | .993 | 1 | .525 | .343 | .910 | .102 | .945 | .655 |
| **AS** | 1 | .998 | 1 | 1 | 1 | 1 | 1 | 1 | .461 | .609 | .434 | .505 | .052 |
| **REC** | 1 | 1 | .737 | 1 | 1 | 1 | 1 | 1 |  | .206 | .027 | .644 | .228 |
| **LEC** | 1 | 1 | .997 | 1 | 1 | 1 | 1 | 1 | 1 | 1 | .168 | .226 | .111 |
| **BG** | 1 | 1 | 1 | 1 | 1 | 1 | 1 | 1 | .861 | 1 | 1 | .312 | .873 |
| **L** | 1 | 1 | .381 | 1 | .076 | 1 | 1 | 1 | 1 | 1 | 1 | 1 | .054 |
| **SM** | 1 | 1 | 1 | 1 | 1 | 1 | 1 | .979 | 1 | 1 | 1 | .982 | 1 |
| **FWER-corrected *p*-value** (typical controls > autism spectrum disorder) | | | | | | | | | | | | | |

**Supplementary Table S9-** t-statistic and effect size (Cohen’s d) and uncorrected and FWER-corrected p-values for females and males group comparison. Uncorrected p-values < .05 and FWER-corrected p-values < .05 are highlighted in grey. AS: anterior salience network; A: auditory network; BG: basal ganglia network; C: cerebellum network; DM: default mode network; HV: high visual network; L: language network; LEC: left executive control network; P: precuneus network; PV: primary visual network; REC: right executive control network; SM: sensorimotor network; VS: visuospatial network.

| **Effect size** (*Cohen’s d*) | | | | | | | | | | | | | |
| --- | --- | --- | --- | --- | --- | --- | --- | --- | --- | --- | --- | --- | --- |
|  | **PV** | **DM** | **A** | **HV** | **VS** | **P** | **C** | **AS** | **REC** | **LEC** | **BG** | **L** | **SM** |
| **PV** | 0.00 | 0.09 | 0.19 | 0.26 | 0.15 | 0.03 | 0.14 | 0.32 | 0.02 | 0.14 | 0.05 | 0.00 | 0.11 |
| **DM** | 0.06 | 0.00 | 0.01 | 0.01 | 0.22 | 0.21 | 0.51 | 0.41 | 0.05 | 0.18 | 0.09 | 0.06 | 0.07 |
| **A** | 1.14 | 0.83 | 0.00 | 0.02 | 0.05 | 0.08 | 0.03 | 0.31 | 0.01 | 0.44 | 0.02 | 0.07 | 0.02 |
| **HV** | 0.12 | 0.71 | 1.80 | 0.00 | 0.02 | 0.21 | 0.16 | 0.16 | 0.13 | 0.05 | 0.02 | 0.07 | 0.05 |
| **VS** | 0.65 | 0.31 | 0.93 | 0.76 | 0.00 | 0.19 | 0.23 | 0.11 | 0.25 | 0.24 | 0.11 | 0.11 | 0.06 |
| **P** | 0.84 | 0.35 | 0.25 | 0.13 | 0.65 | 0.00 | 0.09 | 0.26 | 0.08 | 0.05 | 0.07 | 0.01 | 0.01 |
| **C** | 1.28 | 0.96 | 0.30 | 0.72 | 0.39 | 1.52 | 0.00 | 0.20 | 0.09 | 0.08 | 0.25 | 0.00 | 0.10 |
| **AS** | 0.27 | 1.41 | 1.04 | 0.20 | 0.18 | 0.08 | 0.07 | 0.00 | 0.15 | 0.08 | 0.14 | 0.20 | 0.20 |
| **REC** | 1.27 | 3.48 | 2.10 | 1.00 | 0.09 | 0.16 | 0.40 | 0.10 | 0.00 | 0.18 | 0.03 | 0.06 | 0.21 |
| **LEC** | 0.05 | 2.20 | 1.43 | 0.27 | 0.79 | 1.02 | 1.34 | 0.28 | 0.82 | 0.00 | 0.26 | 0.00 | 0.06 |
| **BG** | 0.06 | 0.30 | 2.37 | 1.45 | 0.46 | 0.56 | 1.26 | 0.17 | 1.94 | 0.96 | 0.00 | 0.11 | 0.04 |
| **L** | 0.14 | 0.47 | 2.51 | 1.29 | 3.12 | 0.26 | 1.63 | 0.03 | 0.36 | 0.76 | 0.49 | 0.00 | 0.13 |
| **SM** | 0.74 | 0.78 | 0.62 | 0.36 | 0.94 | 0.63 | 0.40 | 1.63 | 0.74 | 1.22 | 1.15 | 1.61 | 0.00 |
| ***t*-statistic** | | | | | | | | | | | | | |

| **Uncorrected *p*-value** (females > males) | | | | | | | | | | | | | |
| --- | --- | --- | --- | --- | --- | --- | --- | --- | --- | --- | --- | --- | --- |
|  | **PV** | **DM** | **A** | **HV** | **VS** | **P** | **C** | **AS** | **REC** | **LEC** | **BG** | **L** | **SM** |
| **PV** | 1 | .270 | .086 | .976 | .187 | .495 | .183 | .972 | .511 | .169 | .338 | .492 | .237 |
| **DM** | 1 | 1 | .536 | .577 | .933 | .101 | 1 | .005 | .648 | .101 | .257 | .322 | .669 |
| **A** | .999 | 1 | 1 | .429 | .597 | .295 | .505 | .977 | .544 | .998 | .510 | .725 | .528 |
| **HV** | 1 | 1 | 1 | 1 | .366 | .925 | .160 | .831 | .193 | .411 | .410 | .334 | .355 |
| **VS** | 1 | 1 | 1 | 1 | 1 | .896 | .060 | .783 | .065 | .942 | .749 | .811 | .654 |
| **P** | 1 | .999 | 1 | 1 | 1 | 1 | .284 | .045 | .311 | .615 | .653 | .549 | .542 |
| **C** | 1 | 1 | 1 | 1 | .987 | 1 | 1 | .884 | .289 | .731 | .943 | .465 | .211 |
| **AS** | 1 | .309 | 1 | 1 | 1 | .955 | 1 | 1 | .167 | .329 | .192 | .877 | .915 |
| **REC** | 1 | 1 | 1 | 1 | .991 | 1 | 1 | 1 | 1 | .149 | .555 | .342 | .923 |
| **LEC** | 1 | .999 | 1 | 1 | 1 | 1 | 1 | 1 | 1 | 1 | .957 | .533 | .666 |
| **BG** | 1 | 1 | 1 | 1 | 1 | 1 | 1 | 1 | 1 | 1 | 1 | .248 | .387 |
| **L** | 1 | 1 | 1 | 1 | 1 | 1 | 1 | 1 | 1 | 1 | 1 | 1 | .839 |
| **SM** | 1 | 1 | 1 | 1 | 1 | 1 | 1 | 1 | 1 | 1 | 1 | 1 | 1 |
| **FWER-corrected *p*-value** (females > males) | | | | | | | | | | | | | |

**Supplementary Table S9-** (cont.)

| **Uncorrected *p*-value** (males > females) | | | | | | | | | | | | | |
| --- | --- | --- | --- | --- | --- | --- | --- | --- | --- | --- | --- | --- | --- |
|  | **PV** | **DM** | **A** | **HV** | **VS** | **P** | **C** | **AS** | **REC** | **LEC** | **BG** | **L** | **SM** |
| **PV** |  | .725 | .913 | .023 | .814 | .504 | .819 | .024 | .484 | .825 | .656 | .516 | .769 |
| **DM** | 1 |  | .462 | .424 | .067 | .900 | .001 | .996 | .354 | .898 | .745 | .679 | .330 |
| **A** | 1 | 1 |  | .566 | .403 | .704 | .498 | .023 | .457 | .002 | .491 | .275 | .479 |
| **HV** | .817 | 1 | 1 |  | .624 | .081 | .844 | .173 | .811 | .593 | .586 | .662 | .644 |
| **VS** | 1 | .994 | 1 | 1 |  | .105 | .941 | .220 | .938 | .056 | .253 | .192 | .334 |
| **P** | 1 | 1 | 1 | .997 | 1 |  | .720 | .958 | .695 | .387 | .348 | .455 | .447 |
| **C** | 1 | .024 | 1 | 1 | 1 | 1 |  | .116 | .716 | .270 | .058 | .536 | .792 |
| **AS** | .848 | 1 | .805 | 1 | 1 | 1 | 1 |  | .831 | .674 | .803 | .122 | .086 |
| **REC** | 1 | 1 | 1 | 1 | 1 | 1 | 1 | 1 |  | .852 | .444 | .659 | .075 |
| **LEC** | 1 | 1 | .128 | 1 | .985 | 1 | 1 | 1 | 1 |  | .043 | .464 | .335 |
| **BG** | 1 | 1 | 1 | 1 | 1 | 1 | .986 | 1 | 1 | .954 |  | .753 | .620 |
| **L** | 1 | 1 | 1 | 1 | 1 | 1 | 1 | 1 | 1 | 1 | 1 |  | .164 |
| **SM** | 1 | 1 | 1 | 1 | 1 | 1 | 1 | .999 | .997 | 1 | 1 | 1 |  |
| **FWER-corrected *p*-value** (males > females) | | | | | | | | | | | | | |

**Supplementary Table S10-** t-statistic and effect size (Cohen’s d) and uncorrected and FWER-corrected p-values for females-specific comparison between autism spectrum disorder and typical controls groups. Uncorrected p-values < .05 and FWER-corrected p-values < .05 are highlighted in grey. AS: anterior salience network; A: auditory network; BG: basal ganglia network; C: cerebellum network; DM: default mode network; HV: high visual network; L: language network; LEC: left executive control network; P: precuneus network; PV: primary visual network; REC: right executive control network; SM: sensorimotor network; VS: visuospatial network.

| **Effect size** (*Cohen’s d*) | | | | | | | | | | | | | |
| --- | --- | --- | --- | --- | --- | --- | --- | --- | --- | --- | --- | --- | --- |
|  | **PV** | **DM** | **A** | **HV** | **VS** | **P** | **C** | **AS** | **REC** | **LEC** | **BG** | **L** | **SM** |
| **PV** | 0.00 | 0.00 | 0.24 | 0.10 | 0.32 | 0.34 | 0.16 | 0.04 | 0.43 | 0.05 | 0.19 | 0.25 | 0.35 |
| **DM** | 0.06 | 0.00 | 0.02 | 0.14 | 0.03 | 0.13 | 0.22 | 0.17 | 0.47 | 0.53 | 0.13 | 0.14 | 0.10 |
| **A** | 1.14 | 0.83 | 0.00 | 0.50 | 0.26 | 0.34 | 0.10 | 0.13 | 0.45 | 0.52 | 0.36 | 0.38 | 0.05 |
| **HV** | 0.12 | 0.71 | 1.80 | 0.00 | 0.14 | 0.04 | 0.13 | 0.08 | 0.15 | 0.35 | 0.71 | 0.03 | 0.27 |
| **VS** | 0.65 | 0.31 | 0.93 | 0.76 | 0.00 | 0.14 | 0.05 | 0.04 | 0.13 | 0.36 | 0.14 | 0.68 | 0.32 |
| **P** | 0.84 | 0.35 | 0.25 | 0.13 | 0.65 | 0.00 | 0.37 | 0.15 | 0.01 | 0.45 | 0.27 | 0.22 | 0.33 |
| **C** | 1.28 | 0.96 | 0.30 | 0.72 | 0.39 | 1.52 | 0.00 | 0.05 | 0.27 | 0.15 | 0.07 | 0.25 | 0.19 |
| **AS** | 0.27 | 1.41 | 1.04 | 0.20 | 0.18 | 0.08 | 0.07 | 0.00 | 0.31 | 0.23 | 0.11 | 0.03 | 0.07 |
| **REC** | 1.27 | 3.48 | 2.10 | 1.00 | 0.09 | 0.16 | 0.40 | 0.10 | 0.00 | 0.03 | 0.39 | 0.37 | 0.20 |
| **LEC** | 0.05 | 2.20 | 1.43 | 0.27 | 0.79 | 1.02 | 1.34 | 0.28 | 0.82 | 0.00 | 0.40 | 0.12 | 0.12 |
| **BG** | 0.06 | 0.30 | 2.37 | 1.45 | 0.46 | 0.56 | 1.26 | 0.17 | 1.94 | 0.96 | 0.00 | 0.09 | 0.05 |
| **L** | 0.14 | 0.47 | 2.51 | 1.29 | 3.12 | 0.26 | 1.63 | 0.03 | 0.36 | 0.76 | 0.49 | 0.00 | 0.23 |
| **SM** | 0.74 | 0.78 | 0.62 | 0.36 | 0.94 | 0.63 | 0.40 | 1.63 | 0.74 | 1.22 | 1.15 | 1.61 | 0.00 |
| ***t*-statistic** | | | | | | | | | | | | | |

| **Uncorrected *p*-value** (autism spectrum disorder - females > typical controls - females) | | | | | | | | | | | | | |
| --- | --- | --- | --- | --- | --- | --- | --- | --- | --- | --- | --- | --- | --- |
|  | **PV** | **DM** | **A** | **HV** | **VS** | **P** | **C** | **AS** | **REC** | **LEC** | **BG** | **L** | **SM** |
| **PV** | 1 | .433 | .081 | .499 | .124 | .986 | .696 | .422 | .991 | .539 | .169 | .819 | .063 |
| **DM** | 1 | 1 | .534 | .400 | .646 | .741 | .896 | .840 | .031 | .006 | .260 | .200 | .640 |
| **A** | .998 | 1 | 1 | .004 | .831 | .931 | .238 | .312 | .956 | .971 | .025 | .987 | .424 |
| **HV** | 1 | 1 | .280 | 1 | .147 | .666 | .295 | .613 | .702 | .956 | < .001 | .466 | .065 |
| **VS** | 1 | 1 | 1 | 1 | 1 | .243 | .595 | .515 | .735 | .956 | .737 | 1 | .916 |
| **P** | 1 | 1 | 1 | 1 | 1 | 1 | .959 | .704 | .539 | .013 | .820 | .842 | .939 |
| **C** | 1 | 1 | 1 | 1 | 1 | 1 | 1 | .331 | .873 | .395 | .602 | .163 | .730 |
| **AS** | 1 | 1 | 1 | 1 | 1 | 1 | 1 | 1 | .082 | .160 | .695 | .331 | .720 |
| **REC** | 1 | .900 | 1 | 1 | 1 | 1 | 1 | .998 | 1 | .562 | .906 | .027 | .811 |
| **LEC** | 1 | .397 | 1 | 1 | 1 | .619 | 1 | 1 | 1 | 1 | .950 | .307 | .705 |
| **BG** | 1 | 1 | .812 | .036 | 1 | 1 | 1 | 1 | 1 | 1 | 1 | .720 | .420 |
| **L** | 1 | 1 | 1 | 1 | 1 | 1 | 1 | 1 | .870 | 1 | 1 | 1 | .925 |
| **SM** | .991 | 1 | 1 | .991 | 1 | 1 | 1 | 1 | 1 | 1 | 1 | 1 | 1 |
| **FWER-corrected *p*-value** (autism spectrum disorder - females > typical controls - females) | | | | | | | | | | | | | |

**Supplementary Table S10-** (cont.)

| **Uncorrected *p*-value** (typical controls – females > autism spectrum disorder – females) | | | | | | | | | | | | | |
| --- | --- | --- | --- | --- | --- | --- | --- | --- | --- | --- | --- | --- | --- |
|  | **PV** | **DM** | **A** | **HV** | **VS** | **P** | **C** | **AS** | **REC** | **LEC** | **BG** | **L** | **SM** |
| **PV** |  | .563 | .914 | .497 | .875 | .016 | .294 | .566 | .010 | .463 | .827 | .183 | .937 |
| **DM** | 1 |  | .465 | .597 | .355 | .257 | .100 | .159 | .969 | .993 | .735 | .804 | .357 |
| **A** | 1 | 1 |  | .995 | .173 | .070 | .752 | .682 | .044 | .032 | .975 | .014 | .584 |
| **HV** | 1 | 1 | 1 |  | .860 | .337 | .702 | .383 | .305 | .046 | 1 | .526 | .934 |
| **VS** | 1 | 1 | 1 | 1 |  | .761 | .414 | .477 | .271 | .045 | .257 | < .001 | .085 |
| **P** | .643 | 1 | .994 | 1 | 1 |  | .041 | .294 | .473 | .986 | .176 | .155 | .062 |
| **C** | 1 | .999 | 1 | 1 | 1 | .939 |  | .671 | .126 | .607 | .393 | .839 | .269 |
| **AS** | 1 | 1 | 1 | 1 | 1 | 1 | 1 |  | .915 | .842 | .313 | .674 | .283 |
| **REC** | .492 | 1 | .954 | 1 | 1 | 1 | 1 | 1 |  | .432 | .094 | .970 | .188 |
| **LEC** | 1 | 1 | .882 | .961 | .960 | 1 | 1 | 1 | 1 |  | .051 | .699 | .296 |
| **BG** | 1 | 1 | 1 | 1 | 1 | 1 | 1 | 1 | .999 | .972 |  | .291 | .577 |
| **L** | 1 | 1 | .597 | 1 | .031 | 1 | 1 | 1 | 1 | 1 | 1 |  | .079 |
| **SM** | 1 | 1 | 1 | 1 | .998 | .990 | 1 | 1 | 1 | 1 | 1 | .996 |  |
| **FWER-corrected *p*-value** (typical controls - females > autism spectrum disorder - females) | | | | | | | | | | | | | |

**Supplementary Table S11-** t-statistic and effect size (Cohen’s d) and uncorrected and FWER-corrected p-values for males-specific comparison between autism spectrum disorder and typical controls groups. Uncorrected p-values < .05 and FWER-corrected p-values < .05 are highlighted in grey. AS: anterior salience network; A: auditory network; BG: basal ganglia network; C: cerebellum network; DM: default mode network; HV: high visual network; L: language network; LEC: left executive control network; P: precuneus network; PV: primary visual network; REC: right executive control network; SM: sensorimotor network; VS: visuospatial network.

| **Effect size** (*Cohen’s d*) | | | | | | | | | | | | | |
| --- | --- | --- | --- | --- | --- | --- | --- | --- | --- | --- | --- | --- | --- |
|  | **PV** | **DM** | **A** | **HV** | **VS** | **P** | **C** | **AS** | **REC** | **LEC** | **BG** | **L** | **SM** |
| **PV** | 0.00 | 0.04 | 0.04 | 0.07 | 0.03 | 0.27 | 0.24 | 0.00 | 0.10 | 0.10 | 0.15 | 0.25 | 0.12 |
| **DM** | 0.06 | 0.00 | 0.22 | 0.21 | 0.05 | 0.05 | 0.02 | 0.32 | 0.63 | 0.25 | 0.00 | 0.02 | 0.17 |
| **A** | 1.14 | 0.83 | 0.00 | 0.03 | 0.09 | 0.15 | 0.09 | 0.28 | 0.29 | 0.06 | 0.27 | 0.21 | 0.22 |
| **HV** | 0.12 | 0.71 | 1.80 | 0.00 | 0.01 | 0.18 | 0.14 | 0.08 | 0.35 | 0.33 | 0.25 | 0.39 | 0.22 |
| **VS** | 0.65 | 0.31 | 0.93 | 0.76 | 0.00 | 0.03 | 0.16 | 0.11 | 0.08 | 0.16 | 0.27 | 0.11 | 0.03 |
| **P** | 0.84 | 0.35 | 0.25 | 0.13 | 0.65 | 0.00 | 0.16 | 0.12 | 0.11 | 0.08 | 0.32 | 0.16 | 0.15 |
| **C** | 1.28 | 0.96 | 0.30 | 0.72 | 0.39 | 1.52 | 0.00 | 0.09 | 0.08 | 0.29 | 0.43 | 0.30 | 0.23 |
| **AS** | 0.27 | 1.41 | 1.04 | 0.20 | 0.18 | 0.08 | 0.07 | 0.00 | 0.30 | 0.14 | 0.11 | 0.15 | 0.31 |
| **REC** | 1.27 | 3.48 | 2.10 | 1.00 | 0.09 | 0.16 | 0.40 | 0.10 | 0.00 | 0.21 | 0.37 | 0.39 | 0.01 |
| **LEC** | 0.05 | 2.20 | 1.43 | 0.27 | 0.79 | 1.02 | 1.34 | 0.28 | 0.82 | 0.00 | 0.02 | 0.37 | 0.22 |
| **BG** | 0.06 | 0.30 | 2.37 | 1.45 | 0.46 | 0.56 | 1.26 | 0.17 | 1.94 | 0.96 | 0.00 | 0.02 | 0.37 |
| **L** | 0.14 | 0.47 | 2.51 | 1.29 | 3.12 | 0.26 | 1.63 | 0.03 | 0.36 | 0.76 | 0.49 | 0.00 | 0.21 |
| **SM** | 0.74 | 0.78 | 0.62 | 0.36 | 0.94 | 0.63 | 0.40 | 1.63 | 0.74 | 1.22 | 1.15 | 1.61 | 0.00 |
| ***t*-statistic** | | | | | | | | | | | | | |

| **Uncorrected *p*-value** (autism spectrum disorder - males > typical controls - males) | | | | | | | | | | | | | |
| --- | --- | --- | --- | --- | --- | --- | --- | --- | --- | --- | --- | --- | --- |
|  | **PV** | **DM** | **A** | **HV** | **VS** | **P** | **C** | **AS** | **REC** | **LEC** | **BG** | **L** | **SM** |
| **PV** | 1 | .529 | .405 | .437 | .597 | .149 | .903 | .414 | .274 | .432 | .808 | .130 | .696 |
| **DM** | 1 | 1 | .861 | .224 | .522 | .439 | .533 | .840 | .001 | .268 | .581 | .567 | .775 |
| **A** | 1 | 1 | 1 | .544 | .639 | .131 | .609 | .159 | .896 | .552 | .092 | .902 | .246 |
| **HV** | 1 | 1 | 1 | 1 | .495 | .269 | .314 | .276 | .024 | .088 | .908 | .040 | .853 |
| **VS** | 1 | 1 | 1 | 1 | 1 | .414 | .213 | .581 | .312 | .279 | .099 | .837 | .476 |
| **P** | 1 | 1 | 1 | 1 | 1 | 1 | .649 | .335 | .373 | .788 | .041 | .258 | .256 |
| **C** | 1 | 1 | 1 | 1 | 1 | 1 | 1 | .631 | .279 | .050 | .935 | .094 | .116 |
| **AS** | 1 | 1 | 1 | 1 | 1 | 1 | 1 | 1 | .941 | .736 | .401 | .655 | .958 |
| **REC** | 1 | .086 | 1 | .847 | 1 | 1 | 1 | 1 | 1 | .847 | .924 | .923 | .570 |
| **LEC** | 1 | 1 | 1 | .998 | 1 | 1 | .975 | 1 | 1 | 1 | .382 | .950 | .885 |
| **BG** | 1 | 1 | .998 | 1 | .999 | .952 | 1 | 1 | 1 | 1 | 1 | .556 | .072 |
| **L** | 1 | 1 | 1 | .941 | 1 | 1 | .999 | 1 | 1 | 1 | 1 | 1 | .800 |
| **SM** | 1 | 1 | 1 | 1 | 1 | 1 | 1 | 1 | 1 | 1 | .996 | 1 | 1 |
| **FWER-corrected *p*-value** (autism spectrum disorder - males > typical controls - males) | | | | | | | | | | | | | |

**Supplementary Table S11-** (cont.)

| **Uncorrected *p*-value** (typical controls - males > autism spectrum disorder - males) | | | | | | | | | | | | | |
| --- | --- | --- | --- | --- | --- | --- | --- | --- | --- | --- | --- | --- | --- |
|  | **PV** | **DM** | **A** | **HV** | **VS** | **P** | **C** | **AS** | **REC** | **LEC** | **BG** | **L** | **SM** |
| **PV** | 1 | .462 | .585 | .572 | .404 | .847 | .097 | .582 | .725 | .566 | .188 | .866 | .312 |
| **DM** | 1 | 1 | .142 | .775 | .477 | .559 | .474 | .161 | .998 | .732 | .423 | .430 | .226 |
| **A** | 1 | 1 | 1 | .457 | .359 | .872 | .390 | .845 | .104 | .454 | .911 | .102 | .751 |
| **HV** | 1 | 1 | 1 | 1 | .507 | .728 | .688 | .717 | .974 | .910 | .094 | .962 | .152 |
| **VS** | 1 | 1 | 1 | 1 | 1 | .590 | .790 | .415 | .688 | .724 | .905 | .163 | .524 |
| **P** | 1 | 1 | 1 | 1 | 1 | 1 | .359 | .664 | .617 | .216 | .959 | .735 | .741 |
| **C** | .999 | 1 | 1 | 1 | 1 | 1 | 1 | .368 | .721 | .951 | .064 | .902 | .880 |
| **AS** | 1 | 1 | 1 | 1 | 1 | 1 | 1 | 1 | .058 | .271 | .603 | .357 | .043 |
| **REC** | 1 | 1 | 1 | 1 | 1 | 1 | 1 | .986 | 1 | .156 | .076 | .077 | .426 |
| **LEC** | 1 | 1 | 1 | 1 | 1 | 1 | 1 | 1 | 1 | 1 | .623 | .053 | .116 |
| **BG** | 1 | 1 | 1 | .999 | 1 | 1 | .990 | 1 | .996 | 1 | 1 | .455 | .927 |
| **L** | 1 | 1 | .999 | 1 | 1 | 1 | 1 | 1 | .996 | .978 | 1 | 1 | .197 |
| **SM** | 1 | 1 | 1 | 1 | 1 | 1 | 1 | .947 | 1 | 1 | 1 | 1 | 1 |
| **FWER-corrected *p*-value** (typical controls - males > autism spectrum disorder - males) | | | | | | | | | | | | | |

**Supplementary Table S12-** t-statistic and effect size (Cohen’s d) and uncorrected and FWER-corrected p-values for autism spectrum disorder-specific comparison between female and male groups. Uncorrected p-values < .05 and FWER-corrected p-values < .05 are highlighted in grey. AS: anterior salience network; A: auditory network; BG: basal ganglia network; C: cerebellum network; DM: default mode network; HV: high visual network; L: language network; LEC: left executive control network; P: precuneus network; PV: primary visual network; REC: right executive control network; SM: sensorimotor network; VS: visuospatial network.

| **Effect size** (*Cohen’s d*) | | | | | | | | | | | | | |
| --- | --- | --- | --- | --- | --- | --- | --- | --- | --- | --- | --- | --- | --- |
|  | **PV** | **DM** | **A** | **HV** | **VS** | **P** | **C** | **AS** | **REC** | **LEC** | **BG** | **L** | **SM** |
| **PV** | 0.00 | 0.10 | 0.30 | 0.22 | 0.34 | 0.27 | 0.19 | 0.34 | 0.25 | 0.08 | 0.25 | 0.25 | 0.35 |
| **DM** | 0.06 | 0.00 | 0.09 | 0.04 | 0.25 | 0.11 | 0.63 | 0.47 | 0.13 | 0.33 | 0.14 | 0.11 | 0.03 |
| **A** | 1.14 | 0.83 | 0.00 | 0.27 | 0.14 | 0.17 | 0.07 | 0.42 | 0.09 | 0.65 | 0.04 | 0.18 | 0.09 |
| **HV** | 0.12 | 0.71 | 1.80 | 0.00 | 0.10 | 0.30 | 0.18 | 0.27 | 0.12 | 0.30 | 0.49 | 0.11 | 0.29 |
| **VS** | 0.65 | 0.31 | 0.93 | 0.76 | 0.00 | 0.09 | 0.13 | 0.12 | 0.14 | 0.53 | 0.31 | 0.37 | 0.25 |
| **P** | 0.84 | 0.35 | 0.25 | 0.13 | 0.65 | 0.00 | 0.05 | 0.11 | 0.02 | 0.21 | 0.37 | 0.20 | 0.25 |
| **C** | 1.28 | 0.96 | 0.30 | 0.72 | 0.39 | 1.52 | 0.00 | 0.12 | 0.09 | 0.15 | 0.08 | 0.04 | 0.11 |
| **AS** | 0.27 | 1.41 | 1.04 | 0.20 | 0.18 | 0.08 | 0.07 | 0.00 | 0.47 | 0.25 | 0.03 | 0.11 | 0.06 |
| **REC** | 1.27 | 3.48 | 2.10 | 1.00 | 0.09 | 0.16 | 0.40 | 0.10 | 0.00 | 0.28 | 0.06 | 0.45 | 0.29 |
| **LEC** | 0.05 | 2.20 | 1.43 | 0.27 | 0.79 | 1.02 | 1.34 | 0.28 | 0.82 | 0.00 | 0.44 | 0.26 | 0.01 |
| **BG** | 0.06 | 0.30 | 2.37 | 1.45 | 0.46 | 0.56 | 1.26 | 0.17 | 1.94 | 0.96 | 0.00 | 0.05 | 0.12 |
| **L** | 0.14 | 0.47 | 2.51 | 1.29 | 3.12 | 0.26 | 1.63 | 0.03 | 0.36 | 0.76 | 0.49 | 0.00 | 0.16 |
| **SM** | 0.74 | 0.78 | 0.62 | 0.36 | 0.94 | 0.63 | 0.40 | 1.63 | 0.74 | 1.22 | 1.15 | 1.61 | 0.00 |
| ***t*-statistic** | | | | | | | | | | | | | |

| **Uncorrected *p*-value** (autism spectrum disorder - females > autism spectrum disorder - males) | | | | | | | | | | | | | |
| --- | --- | --- | --- | --- | --- | --- | --- | --- | --- | --- | --- | --- | --- |
|  | **PV** | **DM** | **A** | **HV** | **VS** | **P** | **C** | **AS** | **REC** | **LEC** | **BG** | **L** | **SM** |
| **PV** | 1 | .296 | .063 | .925 | .096 | .949 | .156 | .910 | .934 | .288 | .115 | .837 | .066 |
| **DM** | 1 | 1 | .336 | .650 | .886 | .308 | .999 | .037 | .796 | .033 | .190 | .207 | .543 |
| **A** | .991 | 1 | 1 | .069 | .683 | .830 | .318 | .950 | .622 | .998 | .376 | .815 | .615 |
| **HV** | 1 | 1 | .993 | 1 | .231 | .933 | .231 | .861 | .729 | .911 | .006 | .711 | .062 |
| **VS** | .999 | 1 | 1 | 1 | 1 | .733 | .286 | .681 | .304 | .985 | .927 | .968 | .841 |
| **P** | 1 | 1 | 1 | 1 | 1 | 1 | .617 | .233 | .439 | .092 | .947 | .815 | .883 |
| **C** | 1 | 1 | 1 | 1 | 1 | 1 | 1 | .672 | .688 | .870 | .683 | .537 | .630 |
| **AS** | 1 | .927 | 1 | 1 | 1 | 1 | 1 | 1 | .018 | .135 | .413 | .651 | .647 |
| **REC** | 1 | 1 | 1 | 1 | 1 | 1 | 1 | .684 | 1 | .127 | .527 | .026 | .905 |
| **LEC** | 1 | .914 | 1 | 1 | 1 | .999 | 1 | 1 | 1 | 1 | .987 | .157 | .488 |
| **BG** | 1 | 1 | 1 | .395 | 1 | 1 | 1 | 1 | 1 | 1 | 1 | .389 | .660 |
| **L** | 1 | 1 | 1 | 1 | 1 | 1 | 1 | 1 | .837 | 1 | 1 | 1 | .844 |
| **SM** | .992 | 1 | 1 | .989 | 1 | 1 | 1 | 1 | 1 | 1 | 1 | 1 | 1 |
| **FWER-corrected *p*-value** (autism spectrum disorder - females > autism spectrum disorder - males) | | | | | | | | | | | | | |

**Supplementary Table S12-** (cont.)

| **Uncorrected *p*-value** (autism spectrum disorder - males > autism spectrum disorder - females) | | | | | | | | | | | | | |
| --- | --- | --- | --- | --- | --- | --- | --- | --- | --- | --- | --- | --- | --- |
|  | **PV** | **DM** | **A** | **HV** | **VS** | **P** | **C** | **AS** | **REC** | **LEC** | **BG** | **L** | **SM** |
| **PV** | 1 | .706 | .937 | .072 | .905 | .054 | .845 | .088 | .065 | .702 | .886 | .163 | .936 |
| **DM** | 1 | 1 | .669 | .352 | .118 | .679 | .002 | .962 | .201 | .966 | .806 | .796 | .458 |
| **A** | 1 | 1 | 1 | .932 | .317 | .171 | .681 | .050 | .370 | .003 | .624 | .181 | .392 |
| **HV** | .993 | 1 | 1 | 1 | .771 | .068 | .769 | .139 | .264 | .083 | .993 | .293 | .939 |
| **VS** | 1 | 1 | 1 | 1 | 1 | .272 | .714 | .323 | .696 | .011 | .075 | .034 | .160 |
| **P** | .978 | 1 | 1 | .991 | 1 | 1 | .386 | .765 | .567 | .904 | .055 | .183 | .118 |
| **C** | 1 | .103 | 1 | 1 | 1 | 1 | 1 | .333 | .317 | .132 | .320 | .469 | .368 |
| **AS** | .998 | 1 | .971 | 1 | 1 | 1 | 1 | 1 | .982 | .867 | .583 | .349 | .351 |
| **REC** | .990 | 1 | 1 | 1 | 1 | 1 | 1 | 1 | 1 | .873 | .475 | .972 | .085 |
| **LEC** | 1 | 1 | .130 | .998 | .591 | 1 | 1 | 1 | 1 | 1 | .014 | .842 | .511 |
| **BG** | 1 | 1 | 1 | 1 | .995 | .979 | 1 | 1 | 1 | .656 | 1 | .601 | .345 |
| **L** | 1 | 1 | 1 | 1 | .903 | 1 | 1 | 1 | 1 | 1 | 1 | 1 | .155 |
| **SM** | 1 | 1 | 1 | 1 | 1 | 1 | 1 | 1 | .998 | 1 | 1 | 1 | 1 |
| **FWER-corrected *p*-value** (autism spectrum disorder - males > autism spectrum disorder - females) | | | | | | | | | | | | | |

**Supplementary Table S13-** t-statistic and effect size (Cohen’s d) and uncorrected and FWER-corrected p-values for typical controls-specific comparison between female and male groups. Uncorrected p-values < .05 and FWER-corrected p-values < .05 are highlighted in grey. AS: anterior salience network; A: auditory network; BG: basal ganglia network; C: cerebellum network; DM: default mode network; HV: high visual network; L: language network; LEC: left executive control network; P: precuneus network; PV: primary visual network; REC: right executive control network; SM: sensorimotor network; VS: visuospatial network.

| **Effect size** (*Cohen’s d*) | | | | | | | | | | | | | |
| --- | --- | --- | --- | --- | --- | --- | --- | --- | --- | --- | --- | --- | --- |
|  | **PV** | **DM** | **A** | **HV** | **VS** | **P** | **C** | **AS** | **REC** | **LEC** | **BG** | **L** | **SM** |
| **PV** | 0.00 | 0.07 | 0.10 | 0.29 | 0.02 | 0.34 | 0.09 | 0.30 | 0.29 | 0.20 | 0.11 | 0.25 | 0.12 |
| **DM** | 0.06 | 0.00 | 0.12 | 0.03 | 0.18 | 0.30 | 0.39 | 0.34 | 0.08 | 0.04 | 0.03 | 0.01 | 0.12 |
| **A** | 1.14 | 0.83 | 0.00 | 0.22 | 0.04 | 0.33 | 0.12 | 0.20 | 0.04 | 0.26 | 0.06 | 0.03 | 0.06 |
| **HV** | 0.12 | 0.71 | 1.80 | 0.00 | 0.05 | 0.12 | 0.16 | 0.07 | 0.39 | 0.38 | 0.48 | 0.24 | 0.20 |
| **VS** | 0.65 | 0.31 | 0.93 | 0.76 | 0.00 | 0.29 | 0.36 | 0.08 | 0.34 | 0.02 | 0.09 | 0.17 | 0.12 |
| **P** | 0.84 | 0.35 | 0.25 | 0.13 | 0.65 | 0.00 | 0.21 | 0.42 | 0.15 | 0.36 | 0.22 | 0.18 | 0.24 |
| **C** | 1.28 | 0.96 | 0.30 | 0.72 | 0.39 | 1.52 | 0.00 | 0.28 | 0.24 | 0.00 | 0.44 | 0.04 | 0.32 |
| **AS** | 0.27 | 1.41 | 1.04 | 0.20 | 0.18 | 0.08 | 0.07 | 0.00 | 0.14 | 0.11 | 0.26 | 0.27 | 0.34 |
| **REC** | 1.27 | 3.48 | 2.10 | 1.00 | 0.09 | 0.16 | 0.40 | 0.10 | 0.00 | 0.07 | 0.02 | 0.31 | 0.13 |
| **LEC** | 0.05 | 2.20 | 1.43 | 0.27 | 0.79 | 1.02 | 1.34 | 0.28 | 0.82 | 0.00 | 0.06 | 0.23 | 0.12 |
| **BG** | 0.06 | 0.30 | 2.37 | 1.45 | 0.46 | 0.56 | 1.26 | 0.17 | 1.94 | 0.96 | 0.00 | 0.17 | 0.17 |
| **L** | 0.14 | 0.47 | 2.51 | 1.29 | 3.12 | 0.26 | 1.63 | 0.03 | 0.36 | 0.76 | 0.49 | 0.00 | 0.11 |
| **SM** | 0.74 | 0.78 | 0.62 | 0.36 | 0.94 | 0.63 | 0.40 | 1.63 | 0.74 | 1.22 | 1.15 | 1.61 | 0.00 |
| ***t*-statistic** | | | | | | | | | | | | | |

| **Uncorrected *p*-value** (typical controls - females > typical controls - males) | | | | | | | | | | | | | |
| --- | --- | --- | --- | --- | --- | --- | --- | --- | --- | --- | --- | --- | --- |
|  | **PV** | **DM** | **A** | **HV** | **VS** | **P** | **C** | **AS** | **REC** | **LEC** | **BG** | **L** | **SM** |
| **PV** | 1 | .378 | .365 | .907 | .532 | .047 | .401 | .918 | .069 | .211 | .742 | .148 | .705 |
| **DM** | 1 | 1 | .710 | .457 | .811 | .093 | .971 | .034 | .369 | .517 | .479 | .582 | .698 |
| **A** | 1 | 1 | 1 | .903 | .453 | .044 | .698 | .875 | .428 | .884 | .641 | .463 | .420 |
| **HV** | 1 | 1 | 1 | 1 | .618 | .687 | .247 | .591 | .031 | .042 | .987 | .123 | .856 |
| **VS** | 1 | 1 | 1 | 1 | 1 | .873 | .053 | .739 | .050 | .472 | .311 | .254 | .324 |
| **P** | .968 | .999 | .954 | 1 | 1 | 1 | .127 | .044 | .287 | .959 | .140 | .225 | .151 |
| **C** | 1 | 1 | 1 | 1 | .975 | 1 | 1 | .892 | .099 | .400 | .960 | .418 | .068 |
| **AS** | 1 | .900 | 1 | 1 | 1 | .954 | 1 | 1 | .797 | .697 | .159 | .888 | .941 |
| **REC** | .992 | 1 | 1 | .889 | .972 | 1 | .999 | 1 | 1 | .387 | .563 | .922 | .743 |
| **LEC** | 1 | 1 | 1 | .955 | 1 | 1 | 1 | 1 | 1 | 1 | .597 | .878 | .738 |
| **BG** | 1 | 1 | 1 | 1 | 1 | 1 | 1 | 1 | 1 | 1 | 1 | .233 | .203 |
| **L** | 1 | 1 | 1 | 1 | 1 | 1 | 1 | 1 | 1 | 1 | 1 | 1 | .657 |
| **SM** | 1 | 1 | 1 | 1 | 1 | 1 | .993 | 1 | 1 | 1 | 1 | 1 | 1 |
| **FWER-corrected *p*-value** (typical controls - females > typical controls - males) | | | | | | | | | | | | | |

**Supplementary Table S13-** (cont.)

| **Uncorrected *p*-value** (typical controls - males > typical controls - females) | | | | | | | | | | | | | |
| --- | --- | --- | --- | --- | --- | --- | --- | --- | --- | --- | --- | --- | --- |
|  | **PV** | **DM** | **A** | **HV** | **VS** | **P** | **C** | **AS** | **REC** | **LEC** | **BG** | **L** | **SM** |
| **PV** | 1 | .617 | .647 | .092 | .475 | .952 | .605 | .080 | .933 | .793 | .266 | .849 | .297 |
| **DM** | 1 | 1 | .294 | .540 | .185 | .901 | .033 | .968 | .635 | .482 | .521 | .427 | .305 |
| **A** | 1 | 1 | 1 | .099 | .551 | .960 | .306 | .124 | .570 | .120 | .359 | .525 | .575 |
| **HV** | .999 | 1 | .999 | 1 | .382 | .309 | .759 | .404 | .969 | .957 | .014 | .875 | .145 |
| **VS** | 1 | 1 | 1 | 1 | 1 | .125 | .948 | .265 | .950 | .517 | .698 | .734 | .676 |
| **P** | 1 | 1 | 1 | 1 | 1 | 1 | .874 | .959 | .712 | .041 | .861 | .784 | .854 |
| **C** | 1 | .895 | 1 | 1 | 1 | 1 | 1 | .109 | .899 | .595 | .040 | .586 | .931 |
| **AS** | .998 | 1 | 1 | 1 | 1 | 1 | 1 | 1 | .204 | .308 | .844 | .107 | .059 |
| **REC** | 1 | 1 | 1 | 1 | 1 | 1 | 1 | 1 | 1 | .616 | .438 | .075 | .260 |
| **LEC** | 1 | 1 | 1 | 1 | 1 | .944 | 1 | 1 | 1 | 1 | .414 | .125 | .260 |
| **BG** | 1 | 1 | 1 | .613 | 1 | 1 | .940 | 1 | 1 | 1 | 1 | .762 | .802 |
| **L** | 1 | 1 | 1 | 1 | 1 | 1 | 1 | 1 | .997 | 1 | 1 | 1 | .341 |
| **SM** | 1 | 1 | 1 | 1 | 1 | 1 | 1 | .987 | 1 | 1 | 1 | 1 | 1 |
| **FWER-corrected *p*-value** (typical controls - males > typical controls - females) | | | | | | | | | | | | | |

**Supplementary Table S14-** Effect of the interaction of diagnosis or sex by site, eye state at scan, and age on the functional brain connectivity (FBC) of pairs of resting-state networks (RSNs) shown to differ between ^1^individuals with autism spectrum disorder (ASD) and typical controls (TC) (diagnosis by site, eye state at scan or age interaction); ^2^females and males (sex by site, eye state at scan or age interaction); and ^3^females with ASD and TC females (diagnosis by site, eye state at scan or age interaction). None of the interaction effects were statistically significant on any of the pairs of RSNs FBC at a significance level of p-value < .05.

| **Pair of resting state networks** | **Interaction** |
| --- | --- |
| default mode – right executive control | **diagnosis by site** (using the whole sample) |
|  | *F*(8,149) = 1.70, *p* = .102 |
|  | **diagnosis by eye state at scan** (using the whole sample) |
|  | *F*(1,163) = 0.67, *p* = .416 |
|  | **diagnosis by age** (using the whole sample) |
|  | *F*(1,163) = 1.53, *p* = .218 |
| default mode – cerebellar | **sex by site** (using the whole sample) |
|  | *F*(8,149) = 0.97, *p* = .464 |
|  | **sex by eye state at scan** (using the whole sample) |
|  | *F*(1,163) = 0.15, *p* = .698 |
|  | **sex by age** (using the whole sample) |
|  | *F*(1,163) = 0.71, *p* = .400 |
| visuospatial – language | **diagnosis by site** (using only females) |
|  | *F*(8,64) = 1.13, *p* = .353 |
|  | **diagnosis by eye state at scan** (using only females) |
|  | *F*(1,78) = 0.23, *p* = .634 |
|  | **diagnosis by age** (using only females) |
|  | *F*(1,78) = 0.48, *p* = .489 |
| high visual – basal ganglia | **diagnosis by site** (using only females) |
|  | *F*(8,64) = 0.65, *p* = .733 |
|  | **diagnosis by eye state at scan** (using only females) |
|  | *F*(1,78) = 1.84, *p* = .178 |
|  | **diagnosis by age** (using only females) |
|  | *F*(1,78) = 0.12, *p* = .728 |


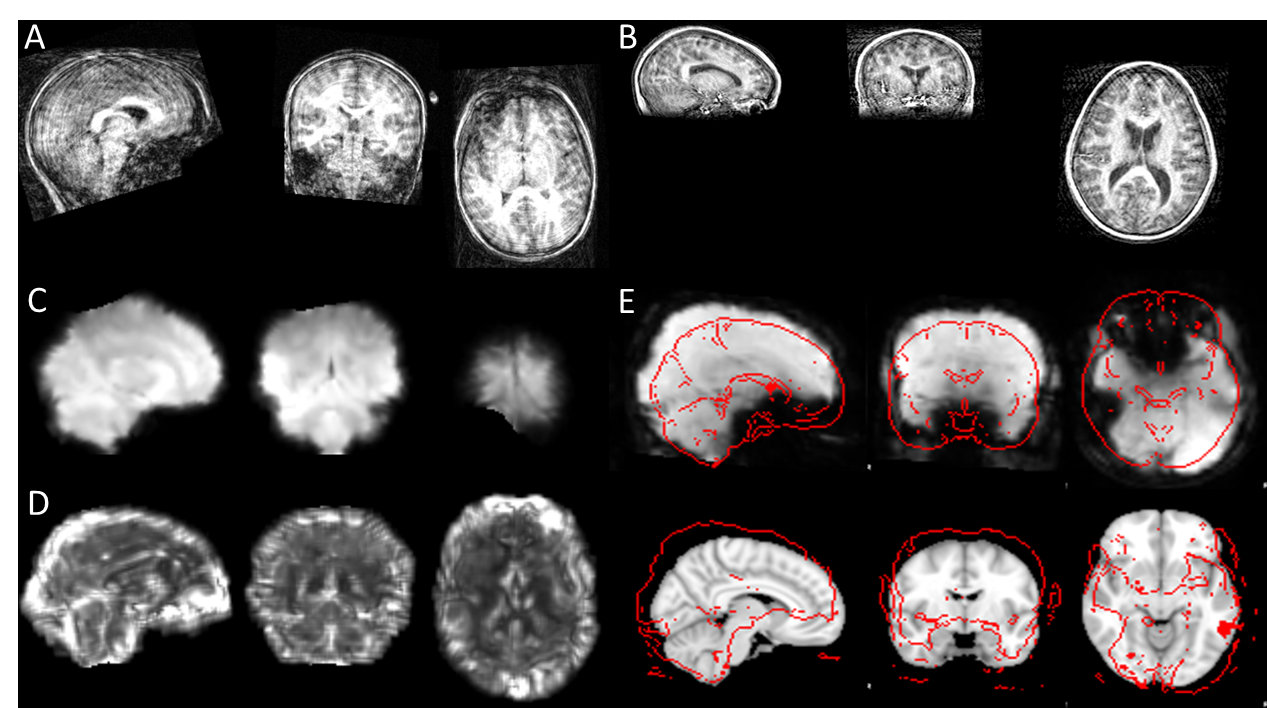


**Supplementary Figure S1**- Examples of images excluded based on qualitative assessment. Exclusion criteria included presence of artifacts in the raw anatomical image (**A** and **B**); lack of brain in the mean preprocessed functional image (**C**); presence of artifacts in the standard deviation of the preprocessed functional data (**D**); and non-successful registration of the functional image to the anatomical one and to the MNI template (**E**).


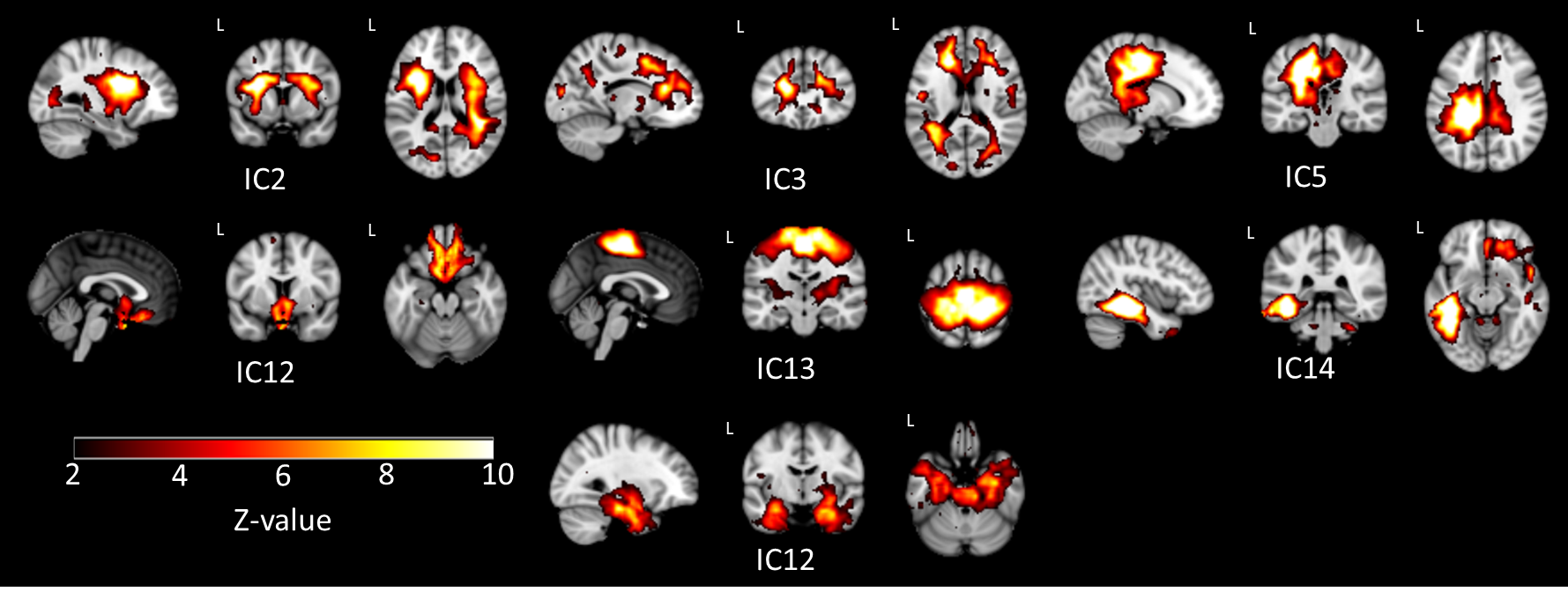


**Supplementary Figure S2-** Spatial configuration the independent components (IC) that were excluded from the analysis.


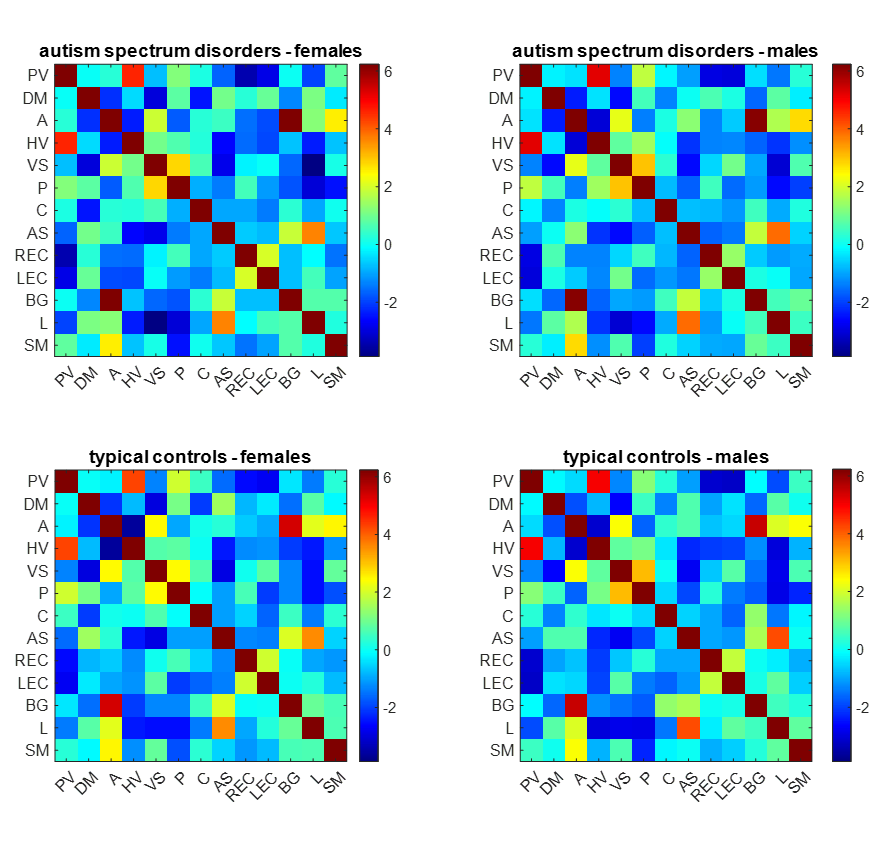


**Supplementary Figure S3-** Averaged functional connectivity matrices with mean z-scored Pearson correlation coefficients for each resting-state network pair, for each analyzed group: autism spectrum disorder – females, autism spectrum disorder – males, typical controls – females and typical controls – males. AS: anterior salience network; A: auditory network; BG: basal ganglia network; C: cerebellum network; DM: default mode network; HV: high visual network; L: language network; LEC: left executive control network; P: precuneus network; PV: primary visual network; REC: right executive control network; SM: sensorimotor network; VS: visuospatial network.
